# CRISPR-dependent endogenous gene regulation is required for virulence in piscine *Streptococcus agalactiae*

**DOI:** 10.1080/22221751.2021.2002127

**Published:** 2021-11-12

**Authors:** Yuhao Dong, Ke Ma, Qing Cao, Hao Huang, Meng Nie, Guangjin Liu, Mingguo Jiang, Chengping Lu, Yongjie Liu

**Affiliations:** a Joint International Research Laboratory of Animal Health and Food Safety, College of Veterinary Medicine, Nanjing Agricultural University, Nanjing, People’s Republic of China; b Guangxi Key Laboratory for Polysaccharide Materials and Modifications, School of Marine Sciences and Biotechnology, Guangxi University for Nationalities, Nanning, People’s Republic of China

**Keywords:** *Streptococcus agalactiae*, CRISPR, virulence, CovR/S, lipoprotein Sag0671

## Abstract

The clustered regularly interspaced palindromic repeats (CRISPR)-Cas (CRISPR-associated) system is a prokaryotic defence against invading mobile genetic elements, such as bacteriophages or exogenous plasmids. Beyond this, this system has been shown to play an important role in controlling the virulence of some bacterial pathogens. *Streptococcus agalactiae* strain GD201008-001, a causative agent of septicemia and meningitis in tilapia, contains a single type II CRISPR-Cas system with Cas9 as a signature protein. In this study, we found that the deletion of CRISPR significantly reduced adhesion, invasion, cytotoxicity and haemolysis, and caused severely attenuated virulence in the piscine *S. agalactiae* strain. RNA-Seq identified 236 endogenous genes regulated by CRISPR, with 159 genes upregulated and 77 genes downregulated. The resulting change in gene transcription by CRISPR was much more pronounced than that by *cas9* in this bacterium, indicating CRISPR-mediated endogenous gene regulation was mostly independently of *cas9*. Subsequent studies showed that CovR/S two-component system was transcriptionally upregulated due to CRISPR deletion, which repressed the expression of the *cylE* gene coding for a cytolytic toxin, and thus decreased the activity of β-haemolysin/cytolysin. However, upregulation of CovR/S was not the contributor to the attenuation phenotype of ΔCRISPR. Further, we demonstrated that CRISPR is capable of repressing the expression of Toll-like receptor 2 (TLR2)-activating lipoprotein Sag0671 and thus dampens the innate immune response. This study revealed that the CRISPR system of *S. agalactiae* exhibited extraordinary potential capability in the regulation of endogenous transcripts, which contributes to bacterial innate immune evasion and virulence.

## Introduction

The clustered regularly interspaced palindromic repeats (CRISPR)-Cas (CRISPR-associated) system is widely distributed in most archaea and many bacteria, which acts as a defense system against invasion by foreign nucleic acids derived from phages, plasmids and viruses [1,2]. The principles and effector module design differentiate the CRISPR-Cas system into two main classes, which further branch into six main types and at least 33 subtypes [3]. CRISPR RNA (crRNA), which harbours the spacer sequence, helps Cas proteins recognize and cleave foreign genetic elements [4]. This cleavage requires a trans-activating crRNA (tracrRNA) to bind with the repeat region of crRNAs via base pairing to form a mature duplex RNA for guidance [5,6]. In addition to the canonical function in immune defense against foreign nucleic acid, the roles of CRISPR-Cas system in bacterial physiology are being uncovered. An increasing number of studies have indicated that CRISPR-Cas is involved in the regulation of endogenous genes, including some genes involved in virulence. The type II-C CRISPR-Cas is indispensable for invasion and replication of *Nesseria meningitidis* in host cells [7]. In *Francisella novicida*, type II-A CRISPR-Cas downregulates the expression of bacterial lipoprotein (BLP) and ultimately promotes both pathogenesis and commensalism [8]. The type I-F CRISPR-Cas system in *Pseudomonas aeruginosa* has been proven to inhibit biofilm formation through crRNA-guided targeting and damaging of integrated prophage DNA [9]. Another study from *P. aeruginosa* [10] showed that CRISPR-Cas system targets the mRNA of the quorum-sensing regulator LasR to evade recognition by Toll-like receptor 4 (TLR4), and consequently diminishes proinflammatory responses and escapes innate immunity.

*Streptococcus agalactiae* or group B *Streptococcus* (GBS) is a Gram-positive zoonotic bacterium that can infect multiple hosts, including humans, bovines and other mammals, and also fish. As a primary pathogen causing meningoencephalitis in cultured tilapia, this bacterium is considered a major threat to the tilapia aquaculture industry [11–13]. Although various virulence factors are known, the exact pathogenesis of this bacterium remains unclear. To date, two different CRISPR-Cas systems have been identified in *S. agalactiae*: Type II-A and I-C [14,15]. Liu et al. [16] reported that the chromosome of *S. agalactiae* strain GD2008-001 only harbours a single type II-A CRISPR-Cas system that consists of four *cas* genes, namely, *cas9, cas1, cas2* and *csn2*, and a CRISPR array with eight spacers. The signature protein Cas9 of type II system has previously been demonstrated to regulate endogenous genes and be involve in the virulence of strain GD2008-001 [17]. Here, we showed that the deletion of CRISPR caused dramatically attenuated virulence in zebrafish and mouse infection models. Further investigation demonstrated that the upregulated CovR/S two-component system is responsible for the decreased haemolytic activity and adhesion, but not the contributor to attenuation phenotype of ΔCRISPR. CRISPR-mediated repression of Toll-like receptor 2 (TLR2)-activating lipoprotein Sag0671 expression is critical for *S. agalactiae* to dampen the host innate response. The findings in the current study advance our understanding of the CRISPR-Cas system function and provide new insights into the contribution of this system to bacterial pathogenesis.

## Materials and methods

### Cell lines, strains, plasmids and growth conditions

RAW264.7 macrophage cells (ATCC) were cultured in high-glucose Dulbecco’s modified Eagle’s medium (DMEM) (Gibco, Grand Island, NY, USA) supplemented with 10% (vol/vol) heat-inactivated foetal bovine serum (FBS) (Gibco, Grand Island, NY, USA). bEnd3 brain endothelial cells (ATCC) were cultured in high-glucose DMEM supplemented with 15% (vol/vol) heat-inactivated FBS.

The bacterial strains and plasmids used in this study are listed in Table S1. The *S. agalactiae* strain GD201008-001, which is β-haemolysin/cytolysin positive and belongs to serotype Ia and multilocus sequence type (MLST) ST-7, was isolated in 2010 from tilapia with meningoencephalitis from a fish farm in Guangdong Province, China [16]. *S. agalactiae* strain GD201008-001 was grown in Todd-Hewitt broth (THB) (Oxoid, Basingstoke, England) or on THB medium with 1.5% (wt/vol) agar. *Escherichia coli* strain DH5α was used as the host for plasmids and cultured in Luria–Bertani (LB) broth or on LB agar medium. The antibiotic spectinomycin (Spc) (Sigma, St. Louis, MO, USA) was added into the solid medium or broth at 100 μg/mL for *S. agalactiae* and 50 μg/mL for *E. coli* when necessary.

### Construction of *S. agalactiae* mutants and complemented strains

To delete the CRISPR array from *S. agalactiae* GD201008-001, a thermosensitive pSET4s suicide vector carrying the homologous CRISPR deletion cassette was constructed. The upstream and downstream arm fragments were first amplified using two sets of primer pairs, CRISPR-A/B and CRISPR-C/D, and then fused into one fragment without the CRISPR cassette by overlap PCR. All primers are listed in Table S2. Both the pSET4s and the fusion fragment were digested by the restriction enzyme *Bam*HI and ligated by the ClonExpress II One Step Cloning Kit (Vazyme, Nanjing, China) to generate the CRISPR deletion vector pSET4s-CRISPR. The pSET4s-CRISPR candidates were transformed into *E. coli* DH5α for propagation, and the construct was verified by colony PCR and sequencing before electroporation into *S. agalactiae* GD201008-001 competent cells, which were selected on THB agar medium with 100 μg/mL Spc [18]. Additional deletion mutants were constructed using the same approach.

To construct the corresponding complementary strain for a deletion mutant, a fragment containing the promoter and complementary locus was amplified and ligated to the pSET2 vector. Then, the recombinant plasmid was electroporated into mutant competent cells. Complementation vector-transformed mutants were cultured on Spc-containing THB agar medium, and positive clones were verified by PCR.

### *In vitro* growth curve assay

Overnight *S. agalactiae* cultures of the wild-type (WT) and its derivative mutant strains were prepared, and the cell densities were equalized by dilution adjustment. Bacterial growth (optical density at 600 nm, OD_600_) in THB were measured every 2 h from 0 h to 12 h after incubation.

### Adhesion assay

The adhesion assay was performed as described previously [19]. bEnd3 brain microvascular endothelial cells were cultured in DMEM supplemented with 15% FBS at 37°C with 5% CO_2_. Cells were seeded in 24-well plates at a density of 10^5^ cells/mL a day before the experiment. Bacterial cells were pelleted at 5000×*g* for 5 min and then resuspended in phosphate-buffered saline (PBS). After washed three times with PBS, the bacterial pellet was resuspended in serum-free DMEM. Cell monolayers were washed three times with PBS prior to being cultured with bacteria at a multiplicity of infection (MOI) of 1:1. Co-cultured cells were incubated at 37°C with 5% CO_2_ for 2 h and washed five times with PBS before being lysed. Lysates were serially diluted in PBS and plated on THB agar medium, and the colony-forming units (CFUs) were counted after overnight incubation at 37°C.

### *S. agalactiae* intracellular survival assay

RAW264.7 macrophages were cultured in DMEM with 10% FBS at 37°C with 5% CO_2_. RAW264.7 cells at a density of 10^5^ cells/mL were seeded in 24-well plates a day before the experiment. Bacterial and cell monolayers were processed in the same way as described for the adhesion assay. Co-cultured cells were incubated at 37°C for 1 h. Extracellular bacteria were removed by washing with PBS five times, refilling the wells with 100 μg/mL penicillin G-containing 1% FBS-DMEM and incubating at 37°C with 5% CO_2_ for 1 h, which represented the 0 h time point. After 2, 4, 6, 8 and 12 h, monolayer cells were washed and lysed. The lysates were serially diluted in PBS and plated on THB agar medium to count the CFUs after incubation at 37°C overnight.

### Cytotoxicity assay

A lactate dehydrogenase (LDH) cytotoxicity assay was performed as previously described [20]. The CytoTox 96 Non-Radioactive Cytotoxicity Assay (Promega, Madison, WI, USA) was utilized to measure the LDH activity. Bacteria were cultured and diluted as described above. RAW264.7 macrophages cultured in 96-well plates were infected with 100 μL of bacterial suspension at an MOI of 1:1 and incubated for 4 h at 37°C with 5% CO_2_. Cells were lysed with Triton X-100 at a final concentration of 1% (vol/vol) as the maximum-release positive control. LDH released by untreated cells and bacteria was measured as the spontaneous-release control. The LDH release value (OD_492_) was measured by a microplate reader. The percentage of cell cytotoxicity was calculated as 100 × [(sample LDH release- spontaneous LDH release)/(maximum LDH release-spontaneous LDH release)], as shown in the manufacturer's protocol.

### LD_50_ determination in zebrafish

The zebrafish used in this study were raised for over a week before being challenged, and their care and feeding were performed according to established protocols [21]. Before being injected into the zebrafish, bacterial cells in late log phase in THB were washed and resuspended in PBS. Zebrafish were anaesthetized with 90 mg/L tricaine methanesulphonate (MS-222) and were then intraperitoneally (*i.p*.) injected with 20 μL of 10-fold serially diluted suspensions of bacteria (10–10^6^ CFU/mL). Each treatment group included 11 zebrafish. Fish in the control group were injected with an equal volume of PBS. Mortality was recorded twice per day for the next 7 days. The 50% lethal dose (LD_50_) values were calculated by the Reed-Muench method [22].

### Murine infection

For the bacterial burden assay, female BALB/c mice (5 to 7 weeks of age) were purchased from the Experimental Animal Center of Yangzhou University. Mice were challenged with 5×10^2^ CFU of the indicated strains. Each treatment group had 6 mice. At 16 h post-infection, brain, spleen and blood samples were harvested, weighed and homogenized in PBS. Homogenates were serially diluted and plated to enumerate the CFUs. For survival experiments, groups of 10 mice were infected *i.p.* with 5 × 10^2^ CFU of the indicated strains and monitored for death every 4 h until 7 days post-infection.

### Detection of blood brain barrier (BBB) opening

To investigate the effect of CRISPR on BBB opening, we used a BALB/c mouse model based on the intravenous injection of β-galactosidase-positive *E. coli* M5 as an indicator. This investigation was carried out as described previously [23]. *S. agalactiae* strains at mid-log growth phase were washed twice in PBS and resuspended in PBS to 1 × 10^3^ CFU/mL. The concentration of *E. coli* M5 was adjusted to 2 × 10^9^ CFU/mL. Three groups of mice were infected with 100 µL of the indicated strains by intraperitoneal injection. At 3, 9, and 15 h post-infection, five mice from each group were selected randomly and inoculated with 100 µL of *E. coli* M5 by the intravenous route. At 5 min post-inoculation with *E. coli* M5, the brains were aseptically removed and homogenized in PBS. Then, the cells were serially diluted and spread onto M63 plates for *E. coli* M5 counting. The bacteria were counted and reported as CFU/g per mouse.

### Transcriptomic analysis

The VAHTSTM mRNA-seq v2 Library Prep Kit for Illumina® (Vazyme, Nanjing, China) was used to generate the transcriptome library for RNA sequencing. Transcriptome reads were mapped against the reference sequence of *S. agalactiae* GD201008-001 using TopHat2 software. Cuffdiff program was used to identify differentially expressed genes (DFGs). DFGs were identified as those with a *P* value <0.05 and a fold-change of >2 between two samples.

### Real-time quantitative PCR (qRT-PCR)

qRT-PCR was carried out as described previously [24]. Total RNA from bacterial cultures at mid-log phase was extracted with an E.Z.N.A. Total RNA Kit I (Omega, Norcross, GA, USA) and then reverse transcribed to cDNA using HiScript II QRT Supermix (Vazyme, Nanjing, China). Two-step relative qRT-PCR was used to measure the mRNA transcription level. The 16S rRNA housekeeping gene was used as the internal control. The primers used for qRT-PCR assays are listed in Table S2. SYBR Green PCR was performed in triplicate using SYBR FAST qPCR Master Mix (KAPA, Boston, MA, USA) following the manufacturer’s protocol on an ABI 7500 RT–PCR system. Changes in gene transcription were determined using the comparative cycle threshold (2^-ΔΔCT^) method [25].

### Haemolytic activity

The haemolysin assay was performed as described previously [26]. Bacterial cells in mid-log phase were pelleted by centrifugation at 3000×*g*, washed with PBS twice and resuspended in 1 mL of PBS with 0.2% glucose. The bacterial suspension (0.1 mL) was pipetted into the first row of a 96-well conical bottom plate, and serial twofold dilutions in PBS with 0.2% glucose from 1:2 to 1:256 were then prepared, each in a final volume of 0.1 mL. Glucose (0.2% in PBS) and 0.1% sodium dodecyl sulphate (SDS) alone were used as negative and positive controls, respectively. An equal volume of washed 1% tilapia red blood cells in 0.2% glucose-containing PBS was then added to each well, and the plate was incubated at 37°C with 5% CO_2_ for 1 h. After incubation, the plate was centrifuged at 3000×*g* for 10 min, and 0.1 mL of the supernatant was transferred to a new plate. Haemoglobin was assessed by measuring the OD_420_ in a spectrophotometer. The reciprocal of the greatest dilution of the supernatant from a given strain that showed at least 50% lysis compared to the SDS control was taken as the haemolytic titre.

### Cytokine assay

RAW264.7 cells were grown in DMEM containing 15% FBS in 24-well tissue culture plates. The monolayers were washed with sterile 10 mM PBS to remove unattached cells. *S. agalactiae* was grown overnight in THB medium at 37°C and washed three times with PBS. The collected bacteria were diluted to 4 × 10^7^ CFU/mL. To inactivate TLR2 signalling, the cells were incubated with 100 μg/mL antagonist C29 for 1 h. The macrophage cells were infected at an MOI of 1:1 for 2 h. The extracellular bacteria were removed by washing the monolayers with PBS and replaced with DMEM containing 100 μg/mL penicillin G. To measure the cytokine expression, the infected cells were sampled at 8 and 16 h after the addition of antibiotics, and treated with 0.02% Triton X-100 for 15 min at 37°C. Uninfected RAW264.7 cells in medium served as controls. The levels of IL-6, IL-1β and TNF-α in macrophages were measured by qRT-PCR. The β-actin housekeeping gene was amplified as an internal control. The primers used for the qRT-PCR assay are listed in Table S2.

### Statistical analysis

Data were analysed with SPSS Statistics version 20.0. Multiple comparisons were performed by analysis of variance (ANOVA) for the qRT-PCR results. The nonparametric Mann–Whitney U test was used for analysis of the data obtained from animal experiments and intracellular assays. A value of *P* < 0.05 indicated a significant difference, and all error bars in the figures represent the standard deviation of independent experiments.

## Results

### Analysis of the CRISPR-Cas systems of *S. agalactiae* GD2008-001

Computational analysis of whole-genome sequence using the CRISPR finder program (https://crispr.i2bc.paris-saclay.fr/Server/) revealed a single type II-A CRISPR-Cas system (spans ∼6.6 kb) in *S. agalactiae* GD2008-001, typically consisting of a CRISPR array and four *cas* genes that are organized in an operon. The CRISPR array contains nine unique spacers of 20–31 bp in length, separated by the eight identical 36-bp repeat sequences. Four *cas* genes are sequentially located upstream of the CRISPR array, including *cas9* (locus_tag: A964_0899), *cas1* (locus_tag: A964_0900), *cas2* (locus_tag: A964_0901), and *csn2* (locus_tag: A964_0902). A tracrRNA sequence is located upstream of the *cas9* gene and is encoded on the opposite DNA strand. The details of the CRISPR system are shown in Figure S1.

### CRISPR deletion significantly decreases *S. agalactiae* adhesion, invasion and cytotoxicity to host cells

**Figure 1.**
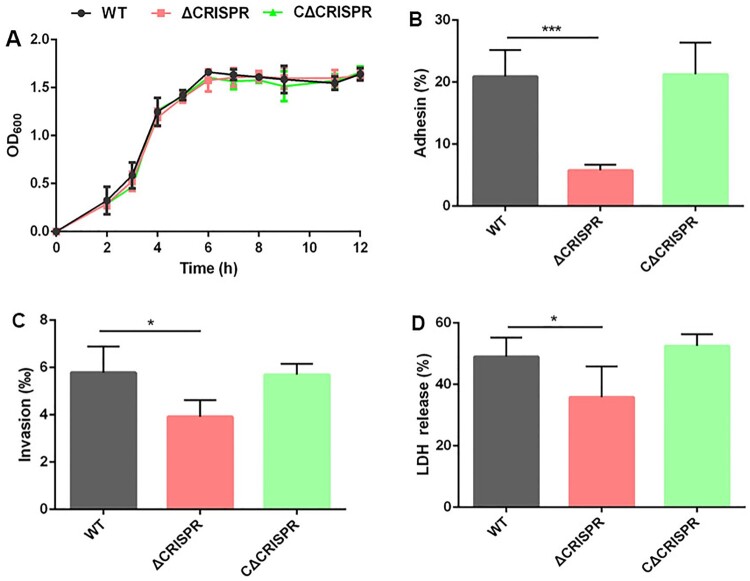
The biological characteristics of the WT and ΔCRISPR strains. (A) Growth curves of the WT and ΔCRISPR strains in THB medium. (B) Adhesion ability of the WT and ΔCRISPR strains to bEnd3 cells. (C) Invasion ability of the WT and ΔCRISPR strains in bEnd3 cells. (D) Cytotoxicity of the WT and ΔCRISPR strains to RAW264.7 macrophage cells. The data are shown as the means ± SD from three independent experiments. *P < 0.05 or ***P < 0.001.

The ΔCRISPR mutant had a similar growth curve as the WT strain in terms of both the growth speed and the highest density at the stationary growth phase when cultured in THB (Figure 1A), suggesting that in nutrient-rich conditions, the deletion of CRISPR did not affect *S. agalactiae* growth. To elucidated the role of CRISPR in bacterial adhesion, we compared the relative level of *S. agalactiae* adhesion to bEnd3 brain microvascular endothelial cells. Compared to the WT strain, ΔCRISPR exhibited decreased adhesion to bEnd3 cells by approximately 4-fold, and the adhesion ability was restored in the complementary strain CΔCRISPR (Figure 1B). Consistent with the bacterial adhesion results, ΔCRISPR also exhibited a 1.5-fold decrease in the invasion rate compared to the WT and CΔCRISPR strains (Figure 1C). Additionally, CRISPR was necessary for *S. agalactiae*-induced macrophage injury. After 4 h of coincubation with *S. agalactiae* strains at an MOI of 1:1, the cytotoxicity of ΔCRISPR on RAW264.7 cells was 1.4-fold lower compared to the WT strain (Figure 1D). Taken together, our results clearly demonstrated the importance of CRISPR in *S. agalactiae* colonization and its induced host cell injury.

### CRISPR is positively involved in *S. agalactiae* virulence and contributes to BBB penetration *in vivo*

**Figure 2.**
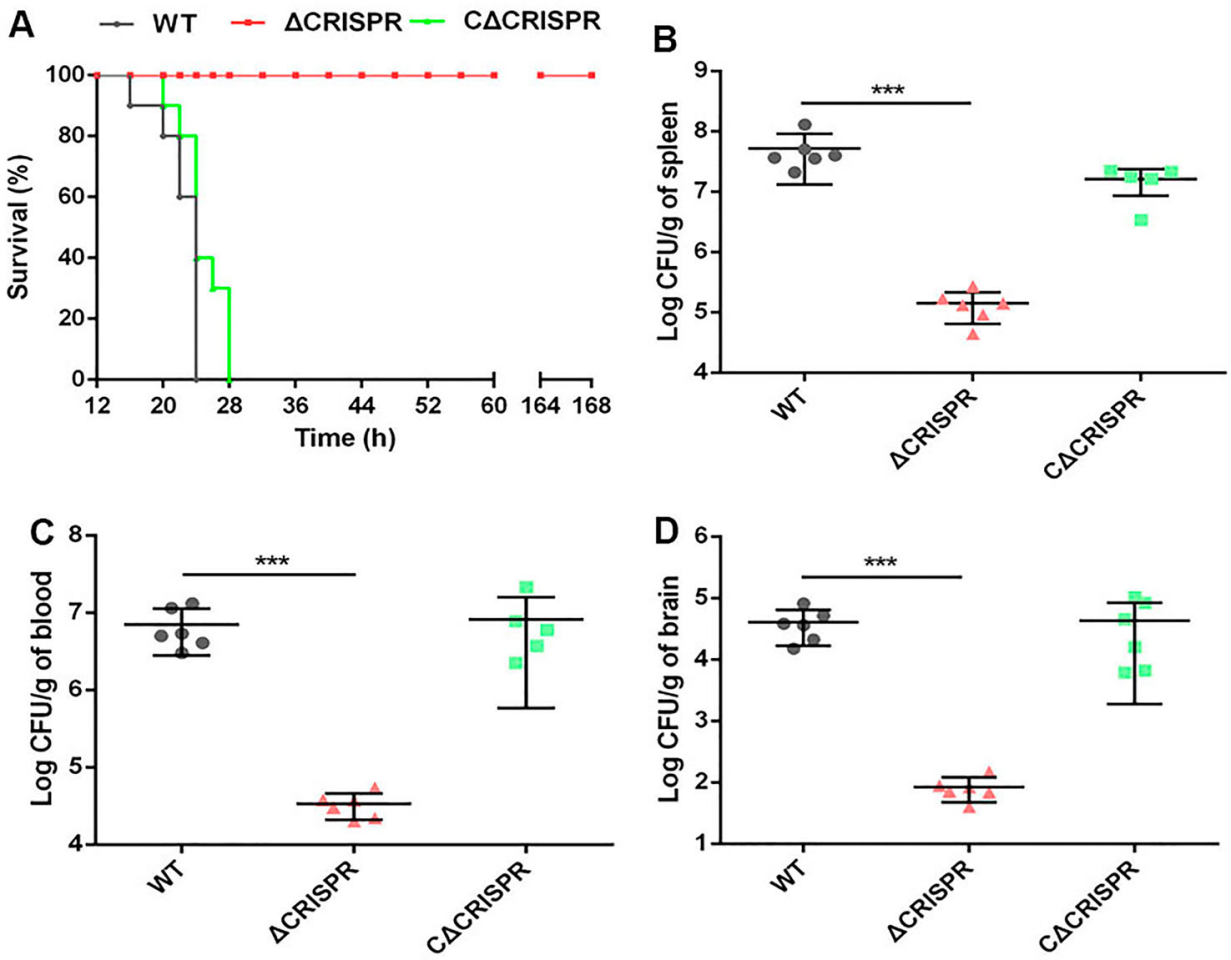
Survival percentage and bacterial distribution in different organs of mice infected with the WT, ΔCRISPR and CΔCRISPR strains. (A) The survival of the mice was monitored every 4 h. At 16 h post-infection, spleen (B), blood (C) and brain (D) were harvested to count the number of CFUs. Bars represent the means for six infected mice. The data are shown as the means ± SD. ***P < 0.001.

**Figure 3.**
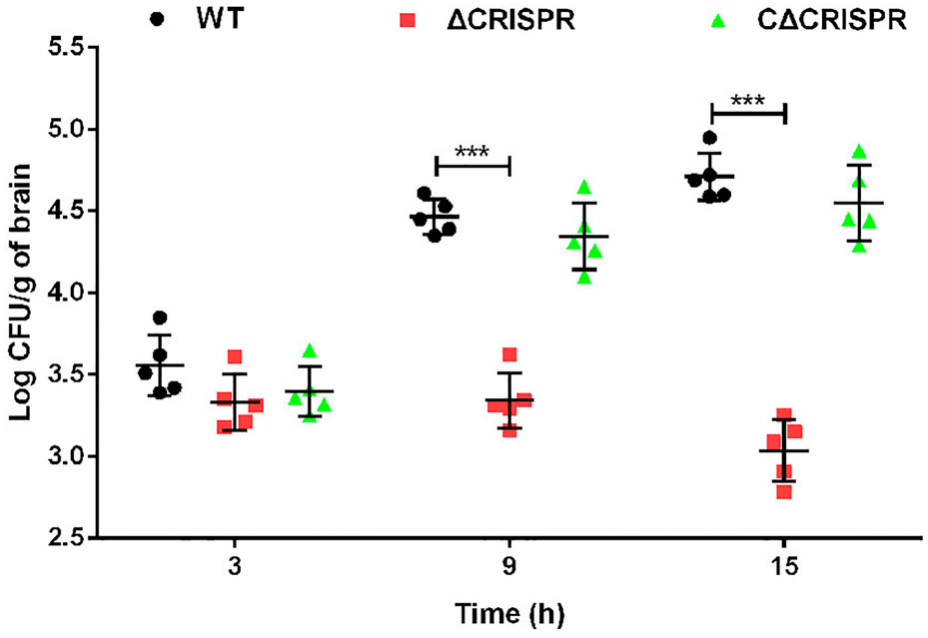
CRISPR promotes BBB penetration. BALB/c mice were intraperitoneally injected with 100 CFU of the WT, ΔCRISPR and CΔCRISPR strains. At 3, 9 and 15 h post-infection, the brains were collected. The level of BBB penetration was assessed by quantitative measurement of E. coli M5 loads per gram of brain. ***P < 0.001.

To investigate the role of CRISPR in *S. agalactiae* virulence, zebrafish were injected *i.p*. with the WT, ΔCRISPR or CΔCRISPR strains. The LD_50_ value of the ΔCRISPR strain (1.72 × 10^4^ CFU) was 71-fold higher than that of the WT strain (2.43 × 10^2^ CFU), which was restored to 5.46 × 10^2^ CFU after complementation with CRISPR (Table S3). Furthermore, we tested mortality in infected mice. Mice infected with the WT or CΔCRISPR strains rapidly succumbed to death, with 100% mortality within 28 h after injection. However, ΔCRISPR did not cause any death, even 128 h after infection (Figure 2A). To better understand the effect of CRISPR on the multiplication and distribution of S. *agalactiae* in hosts, the bacterial burdens in the blood, spleen and brain were calculated. At 16 h post-infection, the deletion of CRISPR resulted in significantly decreased bacterial loads in the spleen (368-fold) (Figure 2B), blood (210-fold) (Figure 2C) and brain (433-fold) (Figure 2D). To colonize the brain, *S. agalactiae* must traverse the BBB. We used a BALB/c mouse model to assess the integrity of the BBB. Mice infected with the WT strain exhibited a significantly greater amount of *E. coli* M5 in brains at 9 h post-infection, compared to mice infected with ΔCRISPR, and the increasing trend was more pronounced at 15 h post-infection (Figure 3). CRISPR complementation partially restored the capacity of ΔCRISPR to disrupt the BBB. Thus, the marked defect of ΔCRISPR in colonizing the brain may, at least in part, be explained by the reduced capacity of this strain to penetrate the BBB.

### Identification of the DEGs in ΔCRISPR by RNA sequencing

**Figure 4.**
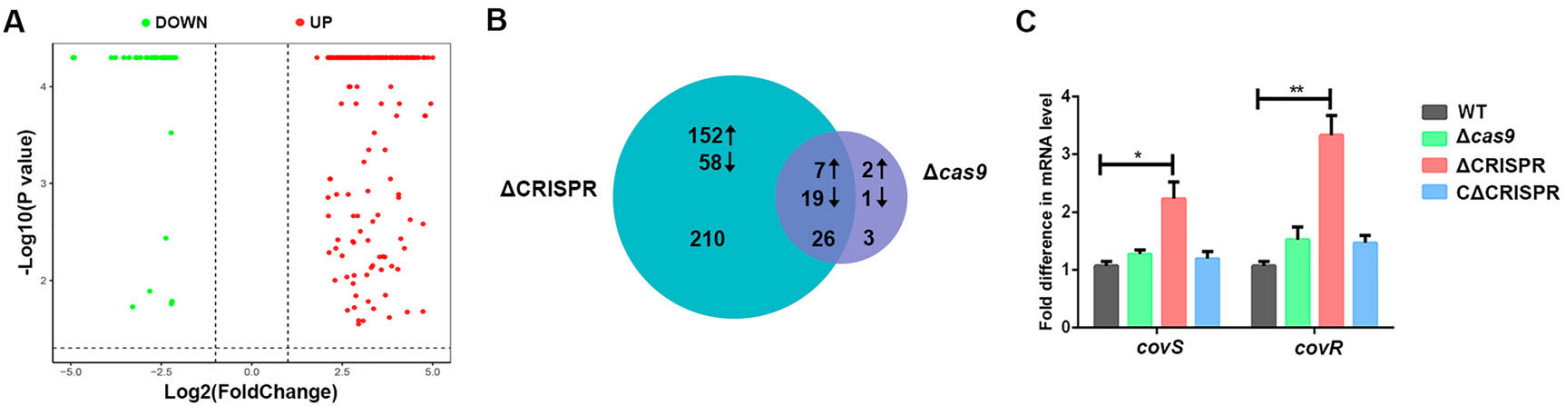
Comparative transcriptome analysis between the WT and ΔCRISPR strains. (A) Volcano plot. The *X*-axis is the log2 of the linear fold change (ΔCRISPR/WT), and the *Y*-axis is the negative log10 of the Benjamini-Hochberg corrected *t*-test *P* value. Up- and down-regulated genes are indicated in shades of red and green, respectively. (B) Venn diagram showing the numbers of differentially abundant genes between ΔCRISPR and Δcas9. (C) The transcription levels of covR/S in the WT, ΔCRISPR, CΔCRISPR and Δcas9 strains. *P < 0.05 or **P < 0.01.

To better understand the mechanisms by which CRISPR influences *S. agalactiae* virulence, we performed transcriptome analysis to compare the differences between the WT and ΔCRISPR strains. A total of 236 DEGs were identified in ΔCRISPR, with 77 genes downregulated and 159 genes upregulated (Figure 4A; Table S4). In order to determine whether there exists a link between the CRISPR array and the *cas9* gene in regulating endogenous gene expression, we compared the transcription profile obtained from ΔCRISPR (236 genes) in this study with that previously reported in the Δ*cas9* (29 genes) [17]. As shown in Figure 4B, there was an overlap of 26 genes, among which 16 genes are located on the lambdaSa04 prophage gene cluster (Table S4). Notably, 210 genes were only identified in ΔCRISPR but not in Δ*cas9*, with 58 genes downregulated and 152 genes upregulated. By comparing the sequences of the 159 upregulated genes in ΔCRISPR on Freiburg RNA platform (https://rna.informatik.uni-freiburg.de/IntaRNA/Input.jsp), we found that mRNAs of 147 genes could partly hybridize with one or more CRISPR spacers (Table S5), including *covS*, a sensor gene of the CovR/CovS (CsrR/CsrS) two-component system that has been suggested to be a negative regulator of bacterial virulence in several studies [27,28]. We quantified the mRNAs of *covR* and *covS* in the WT, Δ*cas9* and ΔCRISPR strains by qRT-PCR. The deletion of *cas9* did not impact the mRNA levels of *covR* and *covS*, but in ΔCRISPR, both *covR* and *covS* were significantly upregulated (Figure 4C), suggesting that *covS* and *covR* might be regulated by CRISPR independent of *cas9*. Sequence alignment of each crRNA spacer with *covS* mRNA showed eight *covS* mRNA regions that may be recognized by the CRISPR-Cas system (Figure S2).

### Decreased haemolysin and adhesion activities in ΔCRISPR are closely related to the CovR/S two-component system

**Figure 5.**
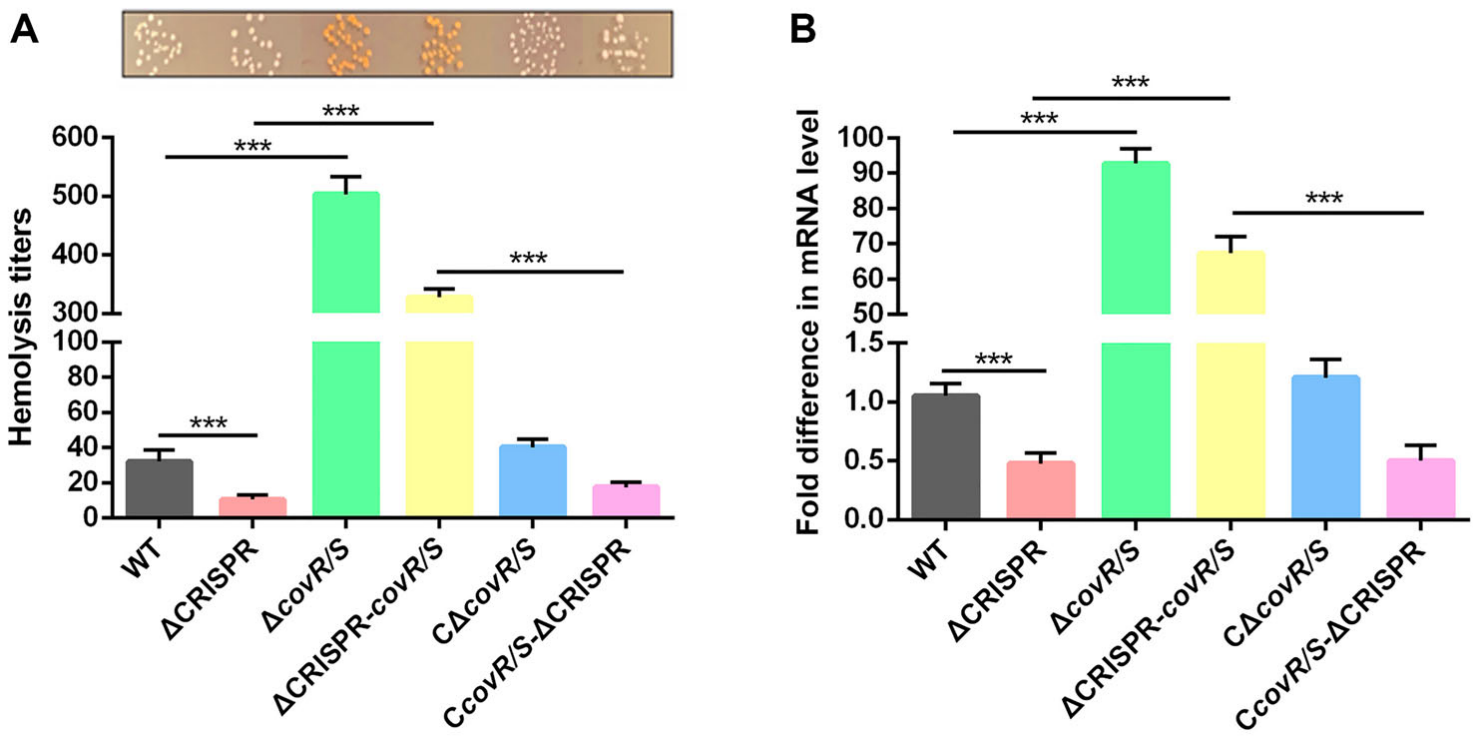
CovR/S is required for the CRISPR-mediated Δ-haemolysin activity decrease. (A) The haemolytic activity of the WT, ΔCRISPR, ΔcovR/S and ΔCRISPR-covR/S strains. (B) The transcription level of cylE in the WT, ΔCRISPR, ΔcovR/S and ΔCRISPR-covR/S strains. ***P < 0.001.

Based on the fact that CovR/S is a well-studied virulence control system in *S. agalactiae* [29] and that it was demonstrated to be downregulated by crRNA in this study, we speculated that CovR/S might have been involved in the repression of virulence in ΔCRISPR. To verify this hypothesis, we deleted the *covR/S* in both the WT and ΔCRISPR strains. As shown in Figure 5A, both the Δ*covR/S* and ΔCRISPR-*covR/S* mutants exhibited increased expression of orange pigment, which changed the colony colour from white to light orange. The amount of pigment produced by GBS always correlates with the amount of haemolysin produced [30]. Compared to the WT strain, ΔCRISPR exhibited 3.2-fold decreased haemolysin activity, but this activity was greatly improved, even higher than that of the WT when *covR/S* was deleted in the ΔCRISPR background. Not surprisingly, Δ*covR/S* showed an over 16-fold increase in haemolytic titre compared to the WT strain, while the haemolytic activity in C*covR/S-*ΔCRISPR was restored to the similar level as in ΔCRISPR. The *cylE* gene has been reported to be necessary for haemolysin production in *S. agalactiae* [31]. Then, we compared the *cylE* transcription level in these strains. Consistent with the haemolysin activity, *cylE* transcription was significantly enhanced in both Δ*covR/S* and ΔCRISPR-*covR/S* but reduced in C*covR/S*-ΔCRISPR as compared with the WT strain (Figure 5B).

**Figure 6.**
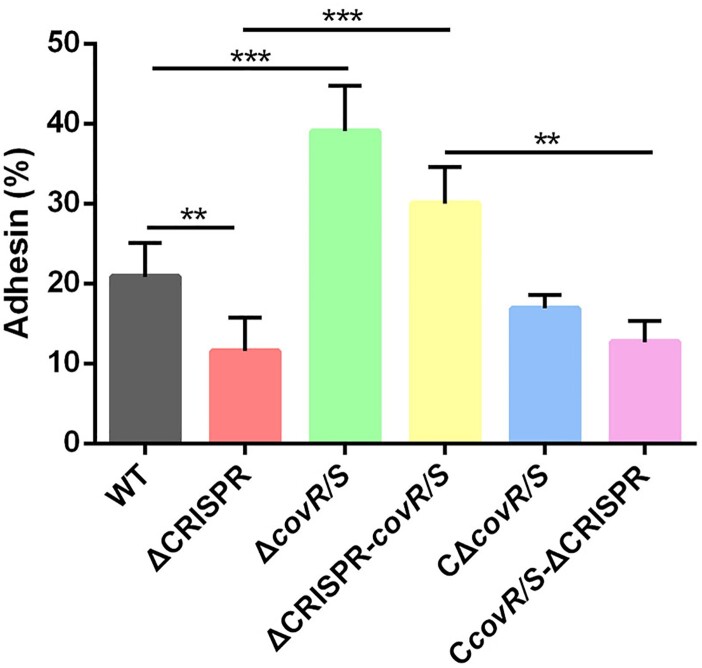
CovR/S is required for the CRISPR-mediated adhesion decrease. Adhesion of the WT, ΔCRISPR, ΔcovR/S and ΔCRISPR-covR/S strains to bEnd.3 cells. At 2 h post-infection, cells were washed and lysed for measurement of the number of CFU. Data are presented as the means ± SD of three independent experiments. **P < 0.01 or ***P < 0.001.

To better evaluate the role of CovR/S in the interaction between *S. agalactiae* and host cells, we compared the bacterial adhesion capacity to bEnd3 endothelial cells. The adhesion rate of Δ*covR/S* was 1.9-fold higher than that of the WT strain. The absence of *covR/S* in ΔCRISPR caused the bacterial adhesion to endothelial cells from a repressed to a 1.5-fold higher level than that caused by the WT, while the adhesive abilities of Δ*covR/S* and ΔCRISPR-*covR/S* were restored to the WT or ΔCRISPR levels after *covR/S* complementation (Figure 6). We speculated that the reduced adhesion in ΔCRISPR was due to the upregulated expression of the CovR/S negative regulator.

### Upregulation of CovR/S is not associated with virulence attenuation in ΔCRISPR

**Figure 7.**
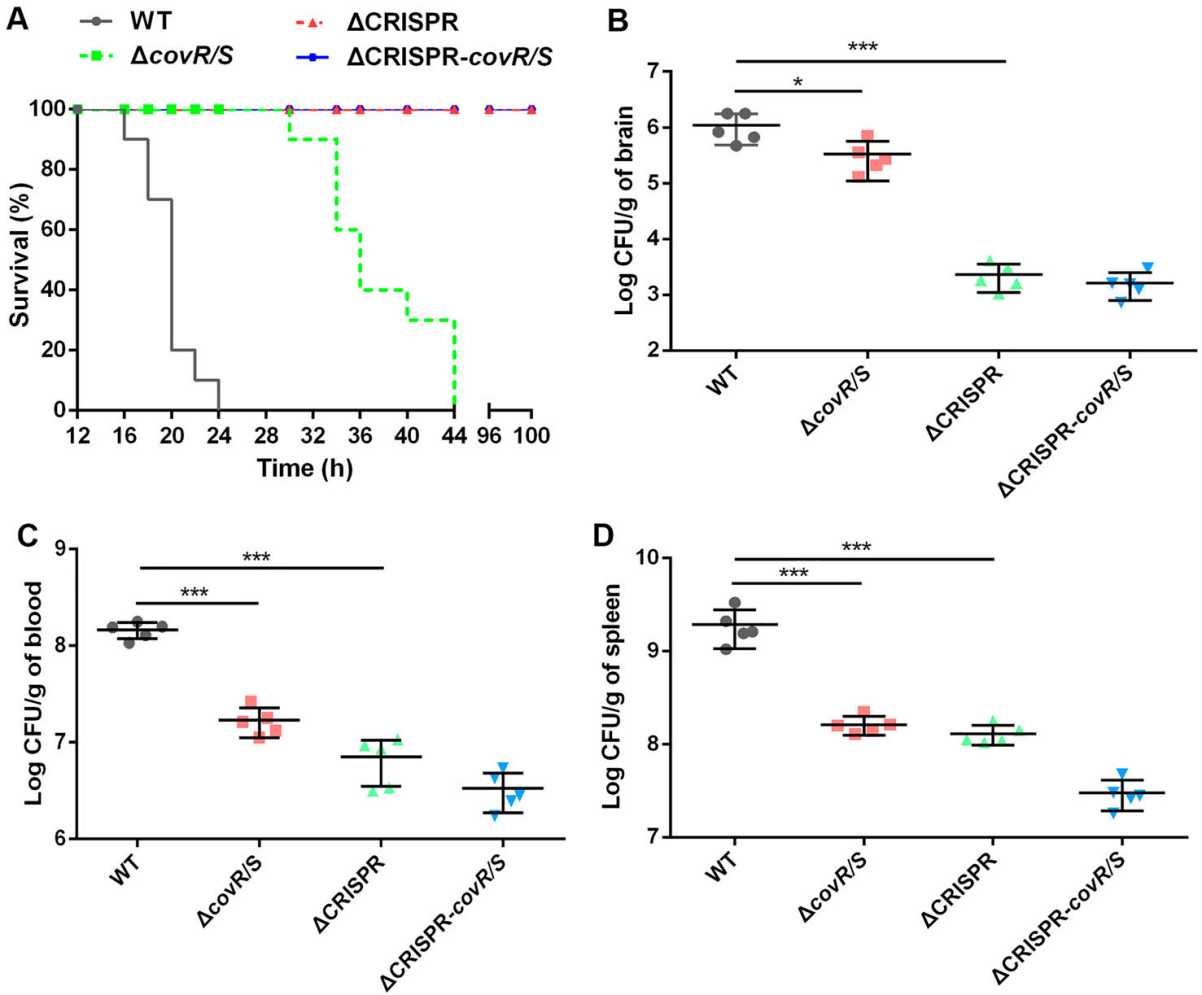
The effect of CovR/S on the CRISPR-mediated attenuation of virulence. (A) Survival percentage of mice infected by the WT, ΔCRISPR, ΔcovR/S and ΔCRISPR-covR/S strains. (B-D) The bacterial distribution in different organs of mice infected by the WT, ΔCRISPR, ΔcovR/S and ΔCRISPR-covR/S strains. *P < 0.05 or ***P < 0.001.

Some previous studies have suggested that haemolysin production and adhesion are essential virulence factors of GBS [31–33]. In this study, we demonstrated that CovR/S acts as a repressor to regulate haemolysin and adhesion activities. Therefore, we assume that the repression of virulence in the ΔCRISPR mutant might be due to CovR/S upregulation. To test this idea, we monitored the mortality rates of WT and its derived mutant strains in mice. Similar to WT, the Δ*covR/S* mutant equally resulted in 100% mortality in the infected mice, but the time of death was 20 h later than that caused by WT (Figure 7A). At 16 h post-infection, colonization by Δ*covR/S* in the brain (Figure 7B), blood (Figure 7C) and spleen (Figure 7D) was lower than that by WT. Furthermore, loss of *covR/S* in the ΔCRISPR background did not become more virulent than ΔCRISPR, as evidenced by similar mortality rate and bacterial loads in tissues. All the data indicated that virulence attenuation in ΔCRISPR could not be interpreted with the upregulated CovR/S.

### Upregulation of the lipoprotein Sag0671 activates TLR2-mediated IL-6 expression in ΔCRISPR

**Figure 8.**
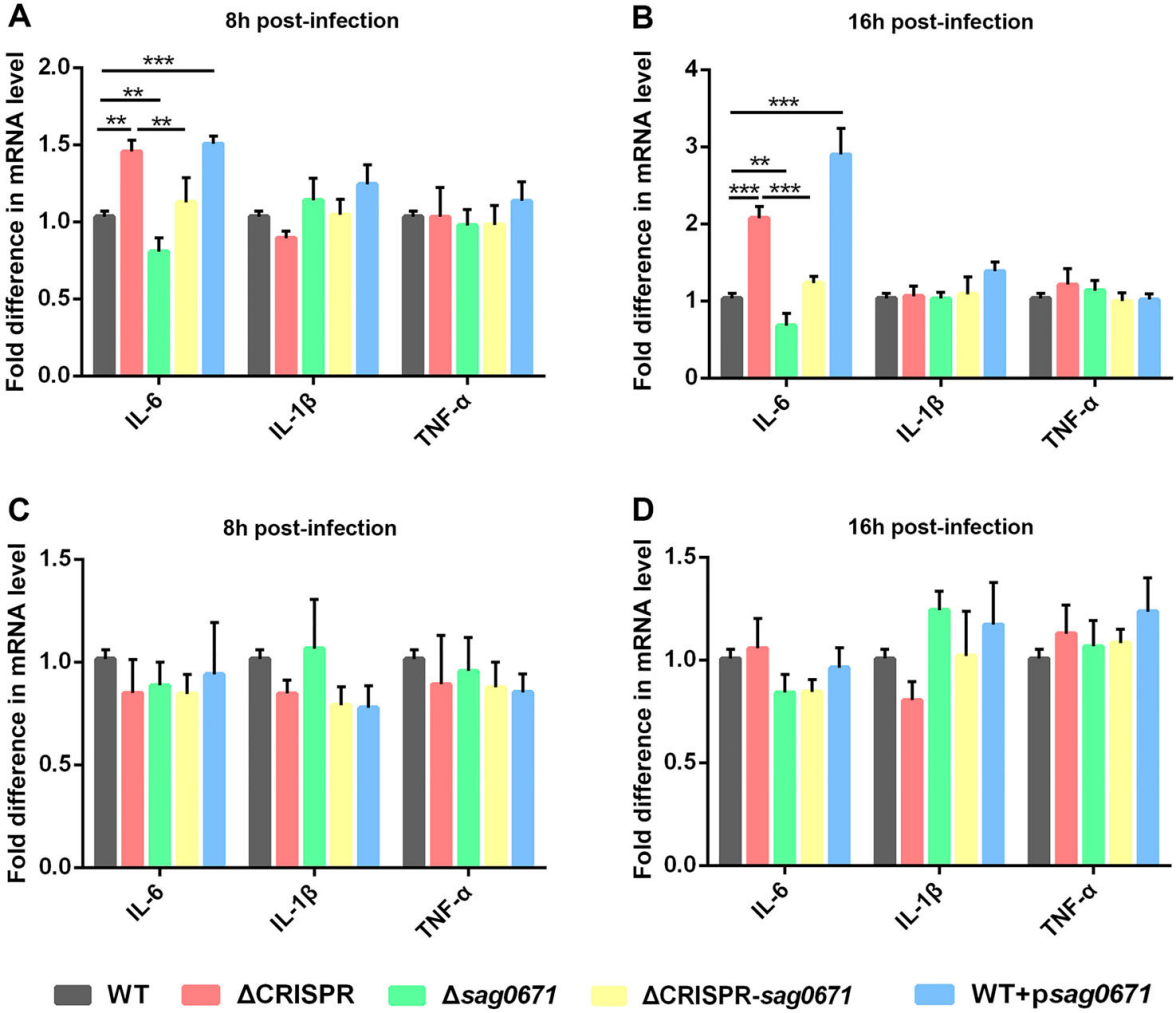
S. agalactiae-induced cytokine expression in RAW264.7 macrophages. (A-B) The expression levels of IL-1β, IL-6 and TNF-α in W264.7 macrophages infected with WT, ΔCRISPR, Δsag0671, WT+psag0671 and ΔCRISPR-sag0671 strains. (C-D) The effect of antagonist C29 on the expression levels of IL-1β, IL-6 and TNF-α in W264.7 macrophages. RAW264.7 cells were grown in DMEM containing 15% foetal bovine serum in 24-well tissue culture plates. For inactivated TLR2 signalling, the cells were incubated with 100 μg/mL antagonist C29 for 1 h. RAW264.7 macrophages were infected with the WT, ΔCRISPR, WT + psag0671 Δsag0671 and ΔCRISPR-sag0671 strains at a MOI of 1:1. Extracellular bacteria were killed by antibiotics, and cells were harvested at 8 and 16 h. The expression levels of IL-1β, IL-6 and TNF-α were measured by qRT-PCR. Data are presented as the means ± SD of three independent experiments. **P < 0.01 or ***P < 0.001.

It has been suggested that lipoprotein can trigger a proinflammatory innate immune response to combat pathogens [34]. Based on our transcriptome data, we found that the expression of the lipoprotein gene *sag0671* was significantly upregulated due to the deletion of CRISPR. The *in silico* analysis predicted that crRNA could partially base pair with the Sag0671 transcript (Figure S3). Next, we detected the expression of IL-6, IL-1β and TNF-α in RAW264.7 cells after infection with the WT or ΔCRISPR strains. As a result, macrophages infected with ΔCRISPR showed an upregulated expression of IL-6 at 8 and 16 h, similar to those with the WT + p*sag0671* strain (overexpression of the *sag0671* gene in the WT strain), while macrophages infected with the Δ*sag0671* mutant expressed lower levels of IL-6 than WT-infected macrophages (Figure 8A, B). In addition, the deletion of *sag0671* in the ΔCRISPR background caused markedly reduced ability of infected macrophages to produce IL-6, similar to that caused by the WT strain, indicating that upregulation of *sag0671* in ΔCRISPR was largely responsible for the high level of IL-6 expression.

Subsequently, to verify whether the increased expression of IL-6 is related to TLR2 which is a host innate immune receptor activated upon sensing bacterial lipoproteins, we disrupted TLR2 signalling by antagonist C29. The data showed that the increased expression of IL-6 in macrophages infected with the ΔCRISPR and WT + p*sag0671* strains was restored to the level seen in WT-infected macrophages after adding the TLR2 inhibitor C29 (Figure 8C, D), suggesting that the increased expression of IL-6 in response to infection by ΔCRISPR was due to hyperstimulation of TLR2. Except for IL-6, no significant difference was observed in the expression of IL-1β and TNF-α among the five groups.

## Discussion

Beyond protection from invading nucleic acids, CRISPR-Cas systems, especially CRISPR-Cas9, have shown an important role in regulating bacterial endogenous genes [35]. However, most of the previous information on the physiological role of CRISPR-Cas9 system comes from studies on Cas9, whereas seldom pay attention to the association of crRNA with bacterial physiology and disease. Considering the link between Cas9 and crRNA, we hypothesize that crRNA may also relate to bacterial virulence. Not surprisingly, our study demonstrated that the deletion of CRISPR caused a dramatic decrease in *S. agalactiae* virulence in challenged zebrafish and mice.

Meningitis is the most common clinical syndrome of *S. agalactiae* infection. The process of penetrating the BBB and invading the central nervous system is essential for the ability of this bacterium to cause meningitis in the host. As the primary elements of the BBB, endothelial cells form capillaries and tight junctions between cells [36,37]. Here, we used bEnd3 brain microvascular endothelial cells to evaluate bacterial adhesion and invasion. As a result, CRISPR deficiency caused significantly reduced bacterial adhesion and invasion to bEnd3 cells, suggesting that CRISPR might be involved in the breaching of the BBB by *S. agalactiae*. Furthermore, we confirmed that CRISPR is necessary for *S. agalactiae* to disrupt BBB integrity using the BALB/c mouse model based on the intravenous injection of β-galactosidase-positive *E. coli* M5 as an indicator.

Next, we wanted to investigate how the CRISPR contributes to bacterial virulence. Transcriptomic RNA-Seq provided more details of the genes impacted by CRISPR. A total of 236 transcriptionally altered genes involved in various physiological processes were identified, suggesting the complex mechanisms via which CRISPR might be involved. After observing an overlap of the 26 DEGs previously identified in Δ*cas9* [17], we hypothesize that CRISPR and *cas9* might be consistently involved in the regulation of these genes. Intriguingly, the *regR* gene, which has previously been reported to be upregulated in Δ*cas9* and negatively regulate *S. agalactiae* virulence by repressing the hyaluronidase activity [17], was also identified among the up-regulated genes in ΔCRISPR. This finding supports the involvement of crRNA-Cas9 complexes in virulence regulation. Notably, however, the virulence attenuation phenotype of ΔCRISPR may not depend entirely on the effect of Cas9, since the decreased virulence in Δ*cas9* is not as proud as that in ΔCRISPR. This idea was further supported by evidence that among 236 DEGs, 210 were only identified in ΔCRISPR, indicating that the regulation of diverse physiological functions mediated by CRISPR is mostly independent of the guide of Cas9. This reminds us of an earlier report in which a CRISPR RNA (originally named RliB) was identified as being involved in the virulence of *Listeria monocytogenes*, despite the absence of *cas* genes [38].

Among the differentially expressed genes that were only identified in ΔCRISPR, the upregulation of *covS* has attracted our attention. CovS is a sensor of the CovR/S (alternate designation CsrR/S) two-component regulatory system, which contributes to bacterial pathogenicity by negatively regulating various genes in *S. agalactiae*, including many virulence factors, such as β-haemolysin/cytolysin, pili, and surface proteins [29,39,40]. In this study, base pairing analysis showed that eight *covS* mRNA regions could be recognized by crRNA spacers, indicating the possibility of direct regulation by CRISPR RNAs. To determine whether there may be any associations among crRNAs, CovR/S and β-haemolysin in *S. agalactiae*, we analysed the *cylE* transcription level and haemolytic activity. Our data suggested that the deletion of CRISPR resulted in remarkably upregulated expression of CovR/S, which repressed the transcription of *cylE* and thus decreased the activity of β-haemolysin/cytolysin. A similar effect was also observed with *in vitro* adhesion of ΔCRISPR to bEnd3 endothelial cells. However, surprisingly, upregulation of CovR/S was not responsible for virulence attenuation of ΔCRISPR, since the deletion of *covR/S* in the WT or ΔCRISPR background did not increase bacterial virulence. This finding indicated that negative regulation on virulence described for CovR/S in most other bacteria appears not to be applicable to CovR/S of *S. agalactiae* strain GD201008-001. We hypothesize that virulence regulation by the CovR/S two-component system may exhibit different discriminatory powers among different bacterial species or strains. This idea is further supported by two early observations: *S. agalactiae* strain A909 with decreased CovR expression showed a dramatically increased capability to cause bloodstream infections and penetration of the BBB [27]; in contrast, inactivation of the CovR/S system in strains 515 and 2603 caused significantly decreased virulence in mice [41].

Previous studies on *F. novicida* have shown that CRISPR-Cas components could downregulate the expression of the lipoprotein FTN_1103 by promoting its mRNA degradation and therefore facilitate bacterial immune evasion [8]. In agreement with this, we found that CRISPR reduced TLR2-dependent expression of the proinflammatory cytokine IL-6 by repressing the lipoprotein Sag0671. IL-6 has been demonstrated to be important for primary resistance to several pathogens [42–44]. Thus, we speculate that CRISPR-mediated suppression of Sag0671 might dampen recognition by TLR2, thus diminishing proinflammatory responses and leading to a virulence-enhanced phenotype. The mechanism of action of CRISPR on Sag0671 is unclear. Notably, however, crRNA partially base pairs with the Sag0671 transcript based on *in silico* prediction. This supports the idea that CRISPR might regulate the expression of lipoprotein Sag0671 via base pairing of the crRNA with the target mRNA, resulting in silencing or degradation of the target transcript. Certainly, we cannot rule out another possibility that CRISPR participates in the regulation of endogenous genes in an indirect way. In *F. novicida*, the CRISPR-Cas system is involved in bacterial pathogenicity by repressing the production of an immunogenic membrane protein via a tracrRNA-based silencing mechanism [8]. In this study, Northern blot analysis demonstrated that the absence of CRISPR could impact the maturation of tracrRNA (Figure S4). We have not investigated whether the tracrRNA was involved in the regulation of endogenous genes in *S. agalactiae* strain GD201008-001. Further studies will be specifically designed to address this issue.

Also, it should be pointed out that as for the attenuated phenotype of ΔCRISPR, the effect of CRISPR deprivation on some regulatory pathways cannot be excluded, since a large number of genes involved in diverse physiological processes (Table S4) were altered. The present investigation together with our previous study of *cas9* [17] suggest that type II-A CRISPR-Cas system plays an important role in *S. agalactiae* virulence by modulating endogenous gene expression. We analyzed the CRISPR/Cas locus among 128 *S. agalactiae* strains with published whole genome sequences using the CRISPR finder program online, and identified four strains with a single type II-A system, in addition to strain GD201008-001 used in this study. BLAST results showed that all the genes that were differentially expressed in the CRISPR array deletion mutant of *S. agalactiae* GD201008-001 could be found in these four strains (Figure S5), implying that endogenous gene regulation mediated by CRISPR RNAs of type II-A might be conserved in *S. agalactiae* strains. Considering that the five bacterial strains analyzed here were isolated from tilapia suffering from streptococcosis in southern China, the significance of this type II-A system in the pathogenesis of piscine *S. agalactiae* may be of great concern.

In conclusion, our work has presented evidence that CRISPR is widely involved in virulence-associated traits in *S. agalactiae*. Although the molecular mechanism of crRNA-involved endogenous gene regulation remains to be clarified, our data provide a rich resource for future studies that may better characterize the CRISPR-Cas function in the regulation of diverse biological characteristics, extending beyond bacterial virulence.

## Supplementary Material

Table_S5.docx

Table_S4_.docx

Table_S3.docx

Table_S2.docx

Table_S1.docx
