# Supplementary material for: CRISPR-dependent endogenous gene regulation is required for virulence in piscine Streptococcus agalactiae
Source: Emerg Microbes Infect. 2021 Nov 12;10(1):2113–24. doi: 10.1080/22221751.2021.2002127 (PMC8592606; doi:10.1080/22221751.2021.2002127)
Supplement: Table_S5.docx [file TEMI_A_2002127_SM5759.docx]

**TableS5 The hybridization of CRISPR spacers with the upregulated genes in ΔCRISPR**

| **Number** | **locus_tag** | **Spacer1** | **Spacer2** | **Spacer3** | **Spacer4** | **Spacer5** | **Spacer6** | **Spacer7** | **Spacer8** |
| --- | --- | --- | --- | --- | --- | --- | --- | --- | --- |
| **Paired** |  |  |  |  |  |  |  |  |  |
| 1 | A964_0003 | - | - | + | - | + | - | - | + |
| 2 | A964_0013 | + | + | + | + | + | - | + | + |
| 3 | A964_0014 | - | + | + | - | + | - | - | + |
| 4 | A964_0019 | + | + | - | + | + | + | - | - |
| 5 | A964_0091 | - | + | + | + | - | - | - | + |
| 6 | A964_0102 | + | + | + | + | + | + | - | + |
| 7 | A964_0115 | + | + | - | - | + | - | + | + |
| 8 | A964_0152 | + | + | - | + | + | - | - | + |
| 9 | A964_0153 | + | + | - | - | + | + | + | + |
| 10 | A964_0188 | + | + | - | - | - | - | + | + |
| 11 | A964_0200 | - | - | - | - | + | - | + | + |
| 12 | A964_0207 | + | + | + | + | + | + | + | + |
| 13 | A964_0208 | - | + | + | + | + | + | - | + |
| 14 | A964_0214 | - | + | + | + | + | + | + | + |
| 15 | A964_0262 | - | + | - | + | - | - | - | + |
| 16 | A964_0264 | + | + | + | - | + | + | + | - |
| 17 | A964_0265 | + | + | - | + | + | - | - | - |
| 18 | A964_0288 | - | - | - | - | + | - | + | + |
| 19 | A964_0310 | - | - | + | - | - | + | + | + |
| 20 | A964_0317 | - | + | + | + | - | + | - | + |
| 21 | A964_0345 | + | - | - | + | + | - | - | + |
| 22 | A964_0351 | + | - | - | - | - | - | + | + |
| 23 | A964_0380 | + | + | + | - | + | + | - | + |
| 24 | A964_0382 | - | - | + | - | - | - | + | + |
| 25 | A964_0394 | + | + | + | - | + | - | - | + |
| 26 | A964_0397 | - | + | + | - | - | + | - | - |
| 27 | A964_0406 | + | - | + | - | - | - | - | + |
| 28 | A964_0414 | - | - | + | - | - | - | - | + |
| 29 | A964_0415 | + | - | + | - | + | + | - | + |
| 30 | A964_0421 | - | + | + | + | - | - | - | + |
| 31 | A964_0437 | - | - | - | + | - | - | - | + |
| 32 | A964_0440 | - | + | - | + | + | + | + | + |
| 33 | A964_0441 | - | + | + | - | + | - | + | + |
| 34 | A964_0507 | - | + | + | - | + | - | + | + |
| 35 | A964_0513 | + | + | + | - | + | + | - | + |
| 36 | A964_0517 | - | - | - | + | + | + | + | - |
| 37 | A964_0552 | + | + | + | + | - | + | - | - |
| 38 | A964_0586 | + | + | + | + | + | + | - | + |
| 39 | A964_0587 | + | + | - | + | + | + | + | + |
| 40 | A964_0591 | + | + | - | + | - | - | - | - |
| 41 | A964_0671 | + | - | - | - | + | + | - | + |
| 42 | A964_0679 | + | - | - | + | + | + | - | + |
| 43 | A964_0687 | - | - | + | - | - | - | - | + |
| 44 | A964_0688 | + | + | - | - | + | + | + | + |
| 45 | A964_0691 | + | + | + | + | + | + | - | + |
| 46 | A964_0708 | + | + | + | + | + | + | + | + |
| 47 | A964_0715 | - | + | + | - | + | + | + | - |
| 48 | A964_0731 | - | + | - | + | + | - | - | + |
| 49 | A964_0745 | + | + | + | + | - | + | + | + |
| 50 | A964_0749 | + | - | + | + | + | + | + | + |
| 51 | A964_0752 | - | + | - | - | + | - | - | + |
| 52 | A964_0754 | + | + | + | - | + | + | + | + |
| 53 | A964_0756 | + | + | - | + | - | + | + | - |
| 54 | A964_0777 | + | + | + | + | + | + | + | - |
| 55 | A964_0785 | + | + | + | + | + | + | + | + |
| 56 | A964_0790 | + | + | + | + | + | - | + | + |
| 57 | A964_0792 | + | + | - | - | + | - | - | + |
| 58 | A964_0803 | + | + | + | + | + |  | + | + |
| 59 | A964_0805 | + | + | + | + | + | + | - | + |
| 60 | A964_0807 | + | + | + | + | + | - | + | + |
| 61 | A964_0816 | + | + | - | + | + | - | - | + |
| 62 | A964_0895 | + | + | + | + | + | + | - | + |
| 63 | A964_0908 | + | + | + | + | - | - | - | + |
| 64 | A964_0909 | - | + | + | - | - | - | - | + |
| 65 | A964_0913 | - | - | + | + | + | - | - | + |
| 66 | A964_0927 | - | + | - | + | + | + | - | + |
| 67 | A964_0929 | + | + | + | + | + | + | - | + |
| 68 | A964_0954 | + | + | + | + | + | + | - | + |
| 69 | A964_1052 | + | + | + | + | - | - | + | + |
| 70 | A964_1060 | + | + | - | - | + | + | + | - |
| 71 | A964_1066 | + | - | + | + | + | - | + | - |
| 72 | A964_1075 | + | + | - | + | + | + | + | - |
| 73 | A964_1081 | + | - | + | - | + | + | - | + |
| 74 | A964_1102 | - | - | + | + | + | - | - | + |
| 75 | A964_1107 | + | - | - | + | + | + | - | + |
| 76 | A964_1118 | + | + | + | + | + | - | + | + |
| 77 | A964_1137 | - | + | + | + | + | + | + | + |
| 78 | A964_1145 | + | + | - | + | - | - | + | + |
| 79 | A964_1146 | + | + | - | + | + | + | + | + |
| 80 | A964_1158 | + | - | - | - | + | + | - | - |
| 81 | A964_1192 | - | + | - | - | + | + | - | + |
| 82 | A964_1250 | + | - | + | - | - | - | - | + |
| 83 | A964_1256 | - | - | + | + | - | - | + | - |
| 84 | A964_1263 | - | + | + | - | + | + | - | + |
| 85 | A964_1270 | + | + | + | + | + | + | - | + |
| 86 | A964_1306 | + | + | - | - | - | - | + | + |
| 87 | A964_1307 | + | + | + | - | + | + | + | + |
| 88 | A964_1309 | - | - | - | - | + | + | - | - |
| 89 | A964_1318 | - | + | - | + | + | - | - | - |
| 90 | A964_1329 | + | + | + | - | - | + | - | - |
| 91 | A964_1367 | + | + | - | + | + | + | + | + |
| 92 | A964_1369 | + | + | + | + | - | + | - | - |
| 93 | A964_1375 | + | + | - | + | + | + | - | + |
| 94 | A964_1390 | - | - | + | - | + | - | + | - |
| 95 | A964_1401 | - | + | - | + | + | + | - | + |
| 96 | A964_1410 | - | - | - | + | - | + | - | - |
| 97 | A964_1415 | + | + | + | + | + | + | + | + |
| 98 | A964_1421 | + | - | - | + | + | - | + | - |
| 99 | A964_1422 | - | - | + | + | + | - | - | + |
| 100 | A964_1440 | - | - | - | + | + | + | + | - |
| 101 | A964_1454 | - | - | - | + | - | - | + | - |
| 102 | A964_1470 | - | - | + | + | + | - | + | + |
| 103 | A964_1473 | - | + | - | - | - | - | - | + |
| 104 | A964_1474 | - | - | - | + | + | - | - | + |
| 105 | A964_1477 | + | + | + | + | + | + | - | + |
| 106 | A964_1509 | - | - | - | + | + | + | + | - |
| 107 | A964_1513 | + | + | + | - | - | - | - | - |
| 108 | A964_1530 | + | + | + | + | + | + | - | + |
| 109 | A964_1531 | + | - | + | + | + | + | - | - |
| 110 | A964_1548 | - | + | + | + | + | + | - | - |
| 111 | A964_1572 | + | + | + | + | + | + | + | + |
| 112 | A964_1577 | + | + | + | + | + | + | + | + |
| 113 | A964_1592 | + | + | + | - | - | + | - | + |
| 114 | A964_1596 | + | + | - | - | + | + | - | + |
| 115 | A964_1597 | - | + | + | - | - | - | - | - |
| 116 | A964_1609 | - | - | + | - | - | + | - | - |
| 117 | A964_1649 | - | + | - | - | + | + | - | + |
| 118 | A964_1655 | + | + | + | - | - | + | - | - |
| 119 | A964_1667 | - | + | - | - | + | - | - | - |
| 120 | A964_1678 | - | - | - | - | - | - | - | - |
| 121 | A964_1710 | + | + | - | + | + | - | + | + |
| 122 | A964_1713 | - | - | + | + | + | + | - | + |
| 123 | A964_1728 | - | + | + | + | - | + | + | + |
| 124 | A964_1764 | + | - | - | + | + | + | + | - |
| 125 | A964_1801 | - | + | + | - | + | - | - | + |
| 126 | A964_1805 | - | - | - | + | + | + | - | - |
| 127 | A964_1819 | - | - | + | - | + | + | - | + |
| 128 | A964_1831 | + | + | - | + | + | - | + | + |
| 129 | A964_1881 | - | - | - | - | - | - | + | - |
| 130 | A964_1899 | - | - | - | + | + | + | - | - |
| 131 | A964_1902 | - | - | - | - | + | + | - | + |
| 132 | A964_1933 | + | + | + | + | + | + | + | + |
| 133 | A964_1959 | - | - | - | + | - | - | - | + |
| 134 | A964_1970 | + | + | + | + | + | + | + | + |
| 135 | A964_1974 | + | + | + | + | + | + | - | + |
| 136 | A964_1975 | + | + | + | - | + | + | - | + |
| 137 | A964_1977 | + | + | + | - | + | + | - | + |
| 138 | A964_1979 | + | + | + | - | + | + | - | + |
| 139 | A964_1981 | + | + | + | + | + | + | + | + |
| 140 | A964_1982 | + | + | - | - | + | - | - | + |
| 141 | A964_1995 | - | + | + | - | - | - | - | - |
| 142 | A964_2000 | - | + | + | + | + | - | + | + |
| 143 | A964_2001 | - | - | - | + | + | + | - | - |
| 144 | A964_2003 | + | + | - | + | + | + | + | + |
| 145 | A964_2016 | + | + | + | - | - | + | - | + |
| 146 | A964_2020 | - | - | + | + | - | - | - | - |
| 147 | A964_2021 | + | + | + | - | + | - | - | + |
| **Unpaired** |  |  |  |  |  |  |  |  |  |
| 1 | A964_0168 | - | - | - | - | - | - | - | - |
| 2 | A964_0390 | - | - | - | - | - | - | - | - |
| 3 | A964_0516 | - | - | - | - | - | - | - | - |
| 4 | A964_0588 | - | - | - | - | - | - | - | - |
| 5 | A964_0823 | - | - | - | - | - | - | - | - |
| 6 | A964_1053 | - | - | - | - | - | - | - | - |
| 7 | A964_1439 | - | - | - | - | - | - | - | - |
| 8 | A964_1528 | - | - | - | - | - | - | - | - |
| 9 | A964_1768 | - | - | - | - | - | - | - | - |
| 10 | A964_1892 | - | - | - | - | - | - | - | - |
| 11 | A964_1912 | - | - | - | - | - | - | - | - |
| 12 | A964_1941 | - | - | - | - | - | - | - | - |
