# Supplementary material for: CRISPR-dependent endogenous gene regulation is required for virulence in piscine Streptococcus agalactiae
Source: Emerg Microbes Infect. 2021 Nov 12;10(1):2113–24. doi: 10.1080/22221751.2021.2002127 (PMC8592606; doi:10.1080/22221751.2021.2002127)
Supplement: Table_S4_.docx [file TEMI_A_2002127_SM5758.docx]

Table S4 The differentially expressed genes in ΔcrRNA compared with wild-type strain

| **Number** | **locus_tag** | **Gene and/or possible function** | **GenBank identification** |
| --- | --- | --- | --- |
| **Upregulated genes** |  |  |  |
| 1 | A964_0003 | Lcb-5, diacylglycerol kinase domain protein | AFS44812.1 |
| 2 | A964_0013 | *mesJ*, tRNA(Ile)-lysidine synthetase | AFS44822.1 |
| 3 | A964_0014 | *hprT*, hypoxanthine-guanine phosphoribosyltransferase | AFS44823.1 |
| 4 | A964_0019 | *recO*, DNA repair protein RecO | AFS44828.1 |
| 5 | A964_0091 | hypothetical protein | AFS44896.1 |
| 6 | A964_0102 | GntR family transcriptional regulator | AFS44907.1 |
| 7 | A964_0115 | pyridine nucleotide-disulfide oxidoreductase | AFS44920.1 |
| 8 | A964_0152 | adaptor protein | AFS44957.1 |
| 9 | A964_0153 | GT_WecA_like gene, glycosyl transferase | AFS44958.1 |
| 10 | A964_0168 | adc operon repressor AdcR | AFS44972.1 |
| 11 | A964_0188 | *ydiL*, Membrane protease | AFS44990.1 |
| 12 | A964_0200 | *lrgA*, Murein hydrolase regulator LrgA | AFS45002.1 |
| 13 | A964_0207 | *treP*, PTS system trehalose-specific transporter subunit IIBCA | AFS45009.1 |
| 14 | A964_0208 | *treC,* alpha amylase | AFS45010.1 |
| 15 | A964_0214 | oxidoreductase, NAD-binding protein | AFS45016.1 |
| 16 | A964_0262 | *rimL*, RimJ/RimL family acetyltransferase | AFS45063.1 |
| 17 | A964_0264 | GNAT family acetyltransferase | AFS45065.1 |
| 18 | A964_0265 | hypothetical protein | AFS45066.1 |
| 19 | A964_0288 | *ccmA*, ABC transporter ATP-binding protein | AFS45089.1 |
| 20 | A964_0310 | *gpsB*, cell division protein GpsB | AFS45111.1 |
| 21 | A964_0317 | *desK*, ATPase, histidine kinase-, DNA gyrase B-, and HSP90-like domain protein | AFS45118.1 |
| 22 | A964_0345 | *yhbH*, ribosomal subunit interface protein | AFS45146.1 |
| 23 | A964_0351 | *acpP*, acyl carrier protein | AFS45152.1 |
| 24 | A964_0380 | *ecsB*, ABC transporter binding protein | AFS45181.1 |
| 25 | A964_0382 | *trmB*, tRNA (guanine-N(7)-)-methyltransferase | AFS45183.1 |
| 26 | A964_0390 | transcriptional repressor CopY | AFS45191.1 |
| 27 | A964_0394 | HAD_Pase, | AFS45195.1 |
| 28 | A964_0397 | *perR*, FUR family transcriptional regulator | AFS45198.1 |
| 29 | A964_0406 | *codA*, cytidine/deoxycytidylate deaminase family protein | AFS45207.1 |
| 30 | A964_0414 | *rnpA*, ribonuclease P protein component | AFS45215.1 |
| 31 | A964_0415 | *yidC*, membrane protein oxaA | AFS45216.1 |
| 32 | A964_0421 | *elaA*, acetyltransferase | AFS45222.1 |
| 33 | A964_0437 | MerR family transcriptional regulator | AFS45234.1 |
| 34 | A964_0440 | aldo/keto reductase oxidoreductase | AFS45235.1 |
| 35 | A964_0441 | cation efflux system protein | AFS45236.1 |
| 36 | A964_0507 | *ftsQ*, cell division protein DivIB | AFS45296.1 |
| 37 | A964_0513 | S4 domain-containing protein | AFS45302.1 |
| 38 | A964_0516 | hypothetical protein | AFS45305.1 |
| 39 | A964_0517 | NUDIX family hydrolase | AFS45306.1 |
| 40 | A964_0552 | *ftsX*, cell division ABC transporter permease FtsX | AFS45337.1 |
| 41 | A964_0586 | sensor histidine kinase VncS | AFS45366.1 |
| 42 | A964_0587 | IS5 family transposase orfB | AFS45367.1 |
| 43 | A964_0588 | CsbD family protein, stress response protein | AFS45368.1 |
| 44 | A964_0591 | HAD superfamily hydrolase | AFS45371.1 |
| 45 | A964_0671 | cross-wall-targeting lipoprotein, sag0671 | AFS45449.1 |
| 46 | A964_0679 | *salY*, ABC-type antimicrobial peptide transport system | AFS45457.1 |
| 47 | A964_0687 | *dedA*, DedA family protein | AFS45465.1 |
| 48 | A964_0688 | *fetA*, ABC transporter ATP-binding protein | AFS45466.1 |
| 49 | A964_0691 | LysR family transcriptional regulator | AFS45469.1 |
| 50 | A964_0708 | *malS*, alpha-amylase | AFS45486.1 |
| 51 | A964_0715 | *hisM*, amino acid ABC transporter permease | AFS45493.1 |
| 52 | A964_0731 | *yraQ*, ABC transporter binding protein | AFS45507.1 |
| 53 | A964_0745 | Acm, U32 family peptidase | AFS45521.1 |
| 54 | A964_0749 | *ribD*, riboflavin biosynthesis protein RibD | AFS45525.1 |
| 55 | A964_0752 | *ribH*, 6,7-dimethyl-8-ribityllumazine synthase | AFS45528.1 |
| 56 | A964_0754 | *yigB*, HAD superfamily hydrolase | AFS45530.1 |
| 57 | A964_0756 | *ebsC*, EbsC family transcriptional regulator | AFS45532.1 |
| 58 | A964_0777 | *metI*, ABC transporter permease | AFS45553.1 |
| 59 | A964_0785 | *comEC*, DNA internalization-related competence protein ComEC/Rec2 | AFS45559.1 |
| 60 | A964_0790 | *holA*, DNA polymerase III subunit delta | AFS45564.1 |
| 61 | A964_0792 | *bglG*, transcriptional antiterminator LicT | AFS45566.1 |
| 62 | A964_0803 | *nagB*, glucosamine-6-phosphate isomerase | AFS45575.1 |
| 63 | A964_0805 | *rsuA*, RNA pseudouridine synthase | AFS45577.1 |
| 64 | A964_0807 | *lysM*, (peptido)glycans binding motif contain protein | AFS45579.1 |
| 65 | A964_0816 | GT8_A4GalT_like gene, glycosyl transferase family protein | AFS45588.1 |
| 66 | A964_0823 | *nrdH*, ribonucleoside-diphosphate reductase 2, NrdH-redoxin | AFS45595.1 |
| 67 | A964_0895 | *hemN*, coproporphyrinogen III oxidase | AFS45665.1 |
| 68 | A964_0908 | *rnaY*, RNaseY, HD superfamily phosphodieaserase | AFS45678.1 |
| 69 | A964_0909 | Pfam00583, acetyltransferase | AFS45679.1 |
| 70 | A964_0913 | LbH_XAT, Xenobiotic acyltransferase | AFS45683.1 |
| 71 | A964_0927 | amino acid ABC transporter, amino acid-binding protein | AFS45697.1 |
| 72 | A964_0929 | *coaA*, pantothenate kinase | AFS45699.1 |
| 73 | A964_0954 | *ftsX*, ABC transporter permease | AFS45722.1 |
| 74 | A964_1052 | *apbE*, ApbE family protein | AFS45820.1 |
| 75 | A964_1053 | NADPH-dependent FMN reductase domain-containing protein | AFS45821.1 |
| 76 | A964_1060 | hypothetical protein | AFS45828.1 |
| 77 | A964_1066 | *yjbM*, RelA/SpoT-type nucleotidyltranferase | AFS45834.1 |
| 78 | A964_1075 | NnaC_like gene, CMP-NeuNAc synthetase | AFS45843.1 |
| 79 | A964_1081 | *potB*, spermidine/putrescine ABC transporter permease | AFS45849.1 |
| 80 | A964_1102 | Ska, Streptokinase-like protein | AFS45867.1 |
| 81 | A964_1107 | *mngR*, GntR family transcriptional regulator | AFS45872.1 |
| 82 | A964_1118 | *alsT*, amino acid carrier protein | AFS45883.1 |
| 83 | A964_1137 | *cpsJ*, capsular polysaccharide biosynthesis protein CpsJ | AFS45902.1 |
| 84 | A964_1145 | *cpsC*, capsular polysaccharide biosynthesis protein CpsC | AFS45910.1 |
| 85 | A964_1146 | *cpsB*, capsular polysaccharide biosynthesis protein CpsB | AFS45911.1 |
| 86 | A964_1158 | *estA*, tributyrin esterase | AFS45923.1 |
| 87 | A964_1192 | nitroreductase family protein | AFS45957.1 |
| 88 | A964_1250 | DUF308, membrane protein, putative | AFS46015.1 |
| 89 | A964_1256 | DegV family protein | AFS46021.1 |
| 90 | A964_1263 | *murN*, beta-lactam resistance factor | AFS46028.1 |
| 91 | A964_1270 | RofA family transcriptional regulator | AFS46035.1 |
| 92 | A964_1306 | hypothetical protein | AFS46071.1 |
| 93 | A964_1307 | ferrichrome ABC transporter ferrichrome-binding protein | AFS46072.1 |
| 94 | A964_1309 | *fecD*, iron compound ABC transporter permease | AFS46074.1 |
| 95 | A964_1318 | *srtA*, sortase | AFS46083.1 |
| 96 | A964_1329 | Glyco_tranf_GTA_type glycosyl transferase | AFS46094.1 |
| 97 | A964_1367 | *secY*, preprotein translocase subunit SecY | AFS46132.1 |
| 98 | A964_1369 | glycosyl transferase family protein | AFS46134.1 |
| 99 | A964_1375 | transcriptional regulator RofA | AFS46140.1 |
| 100 | A964_1390 | *gloA*, lactoylglutathione lyase | AFS46155.1 |
| 101 | A964_1401 | MutR family transcriptional regulator | AFS46166.1 |
| 102 | A964_1410 | *yqfG*, metalloprotease | AFS46175.1 |
| 103 | A964_1415 | *mycA*, myosin-cross-reactive antigen | AFS46180.1 |
| 104 | A964_1421 | *nikE*, peptide ABC transporter ATP-binding protein | AFS46186.1 |
| 105 | A964_1422 | *nikD*, peptide ABC transporter ATP-binding protein | AFS46187.1 |
| 106 | A964_1439 | *mtsB*, manganese ABC transporter ATP-binding protein | AFS46204.1 |
| 107 | A964_1440 | *mtsA*, manganese ABC transporter manganese-binding adhesion liprotein | AFS46205.1 |
| 108 | A964_1454 | *rimI,* Ribosomal protein S18 acetylase | AFS46219.1 |
| 109 | A964_1470 | PTS system lactose/cellobiose family transporter subunit IIC | AFS46233.1 |
| 110 | A964_1473 | *arsC*, ArsC family protein | AFS46236.1 |
| 111 | A964_1474 | *ogt*, methylated-DNA--protein-cysteine S-methyltransferase | AFS46237.1 |
| 112 | A964_1477 | *serC*, Phosphoserine aminotransferase (PSAT) | AFS46240.1 |
| 113 | A964_1509 | biotin repressor family transcriptional regulator | AFS46272.1 |
| 114 | A964_1513 | *acyP*, Acylphosphatase | AFS46276.1 |
| 115 | A964_1528 | *dnaB*, Replication initiation and membrane attachment protein | AFS46289.1 |
| 116 | A964_1530 | sensor histidine kinase CovS | AFS46291.1 |
| 117 | A964_1531 | *csrR*, DNA-binding response regulator | AFS46292.1 |
| 118 | A964_1548 | *tcyK*, ABC transporter binding protein | AFS46309.1 |
| 119 | A964_1572 | *gatA*, PTS system galactitol-specific transporter subunit IIA | AFS46333.1 |
| 120 | A964_1577 | *paaH*, 3-hydroxybutyryl-CoA dehydrogenase | AFS46338.1 |
| 121 | A964_1592 | *manA*, mannose-6-phosphate isomerase | AFS46353.1 |
| 122 | A964_1596 | *scrR*, sucrose operon repressor ScrR | AFS46357.1 |
| 123 | A964_1597 | *nusB*, transcription antitermination protein NusB | AFS46358.1 |
| 124 | A964_1609 | *pepP*, peptidase M24 family protein | AFS46370.1 |
| 125 | A964_1649 | *menA*, 1,4-dihydroxy-2-naphthoate octaprenyltransferase | AFS46410.1 |
| 126 | A964_1655 | PRK09722, allulose-6-phosphate 3-epimerase | AFS46416.1 |
| 127 | A964_1667 | Pfam13302, acetyltransferase | AFS46426.1 |
| 128 | A964_1678 | *rimI*, ribosomal-protein-alanine acetyltransferase | AFS46437.1 |
| 129 | A964_1710 | *dltB*, dltB protein | AFS46469.1 |
| 130 | A964_1713 | DNA-binding response regulator DltR | AFS46472.1 |
| 131 | A964_1728 | *kdgR*, LacI family sugar-binding transcriptional regulator | AFS46487.1 |
| 132 | A964_1764 | RegR family transcriptional regulator | AFS46521.1 |
| 133 | A964_1768 | *marR,* MarR family transcriptional regulator | AFS46525.1 |
| 134 | A964_1801 | *nrdI*, flavoprotein NrdI | AFS46558.1 |
| 135 | A964_1805 | *yesN*, DNA-binding response regulator | AFS46562.1 |
| 136 | A964_1819 | *agrC*, histidine kinase | AFS46574.1 |
| 137 | A964_1831 | *pstS*, phosphate ABC transporter substrate-binding protein | AFS46586.1 |
| 138 | A964_1881 | Pfam13508, acetyltransferase | AFS46634.1 |
| 139 | A964_1892 | Cfa, cAMP factor | AFS46645.1 |
| 140 | A964_1899 | azaleucine resistance protein AzlC | AFS46652.1 |
| 141 | A964_1902 | *ompR*, DNA-binding response regulator | AFS46653.1 |
| 142 | A964_1912 | *rluD*, ribosomal large subunit pseudouridine synthase, RluD subfamily | AFS46663.1 |
| 143 | A964_1933 | membrane protein | AFS46684.1 |
| 144 | A964_1941 | tag, DNA-3-methyladenine glycosylase I | AFS46692.1 |
| 145 | A964_1959 | DNA-binding helix-turn-helix protein | AFS46710.1 |
| 146 | A964_1970 | *baeS*, Signal transduction histidine kinase | AFS46721.1 |
| 147 | A964_1974 | *glnK*, sensor histidine kinase | AFS46725.1 |
| 148 | A964_1975 | *glnL*, response regulator | AFS46726.1 |
| 149 | A964_1977 | *opuB*, ABC-type proline/glycine betaine transport system | AFS46728.1 |
| 150 | A964_1979 | Cpo, Hydrolase, alpha/beta hydrolase fold family | AFS46730.1 |
| 151 | A964_1981 | yhgE, Membrane protein | AFS46732.1 |
| 152 | A964_1982 | prophage Sa05, transcriptional regulator | AFS46733.1 |
| 153 | A964_1995 | MFS, major facilitator family protein | AFS46746.1 |
| 154 | A964_2000 | *pqqL*, Predicted Zn-dependent peptidase | AFS46751.1 |
| 155 | A964_2001 | peptidase M16 inactive domain-containing protein | AFS46752.1 |
| 156 | A964_2003 | *recF*, recombination protein F | AFS46754.1 |
| 157 | A964_2016 | membrane protein | AFS46767.1 |
| 158 | A964_2020 | *rlmH*, rRNA large subunit methyltransferase | AFS46771.1 |
| 159 | A964_2021 | serine peptidase HtrA | AFS46772.1 |
| **Downregulated genes** |  |  |  |
| 1 | A964_0024 | *Purl*, phosphoribosylformylglycinamidine synthase II | AFS44833.1 |
| 2 | A964_0025 | *purF*, amidophosphoribosyl transferase | AFS44834.1 |
| 3 | A964_0026 | *purM*, phosphoribosylaminoimidazole synthetase | AFS44835.1 |
| 4 | A964_0027 | *purN*, phosphoribosylglycinamide formyltransferase | AFS44836.1 |
| 5 | A964_0029 | *purH*, bifunctional phosphoribosylaminoimidazolecarboxamide formyltransferase/IMP cyclohydrolase | AFS44838.1 |
| 6 | A964_0042 | *purD*, phosphoribosylamine--glycine ligase | AFS44851.1 |
| 7 | A964_0043 | *purE* phosphoribosylaminoimidazole carboxylase, catalytic subunit | AFS44852.1 |
| 8 | A964_0044 | *purK*, phosphoribosylaminoimidazole carboxylase ATPase subunit | AFS44853.1 |
| 9 | A964_0047 | DNA-binding protein | AFS44856.1 |
| 10 | A964_0053 | *adhP*, alcohol dehydrogenase | AFS44862.1 |
| 11 | A964_0064 | *rplP*, 50S ribosomal protein L16 | AFS44873.1 |
| 12 | A964_0067 | *rplN*, 50S ribosomal protein L14 | AFS44876.1 |
| 13 | A964_0119 | *rbsC*, ribose ABC transporter permease | AFS44924.1 |
| 14 | A964_0130 | *fbaA*, fructose-bisphosphate aldolase | AFS44935.1 |
| 15 | A964_0210 | *sgaB*, PTS multi-domain regulator | AFS45012.1 |
| 16 | A964_0290 | *sgaB,* PTS system transporter subunit IIB | AFS45091.1 |
| 17 | A964_0362 | *serS,* seryl-tRNA synthetase | AFS45163.1 |
| 18 | A964_0384 | *nusA*, transcription elongation factor | AFS45185.1 |
| 19 | A964_0457 | *gatB*, PTS system galactitol-specific transporter subunit IIB | AFS45250.1 |
| 20 | A964_0597 | Eno, phosphopyruvate hydratase | AFS45377.1 |
| 21 | A964_0605 | collagen-like surface protein | AFS45385.1 |
| 22 | A964_0609 | *dpo*, LambdaSa04, DNA polymerase | AFS45389.1 |
| 23 | A964_0615 | LambdaSa04, DNA primase | AFS45395.1 |
| 24 | A964_0617 | LambdaSa04, SNF2 family helicase | AFS45397.1 |
| 25 | A964_0620 | LambdaW4, DNA methylase | AFS45400.1 |
| 26 | A964_0621 | dcm, LambdaSa04, methyltransferase C-5 | AFS45401.1 |
| 27 | A964_0625 | LambdaSa04, terminase, large subunit | AFS45405.1 |
| 28 | A964_0629 | LambdaSa04, HK97 family portal protein | AFS45409.1 |
| 29 | A964_0630 | *clpP*, LambdaSa04, ClpP endopeptidase | AFS45410.1 |
| 30 | A964_0631 | LambdaSa04, HK97 family major capsid protein | AFS45411.1 |
| 31 | A964_0636 | LambdaSa04, phi13 family major tail protein | AFS45416.1 |
| 32 | A964_0638 | LambdaSa04, TP901 family tail tape measure protein | AFS45418.1 |
| 33 | A964_0639 | LambdaSa04, tail protein | AFS45419.1 |
| 34 | A964_0640 | LambdaSa04, minor structural protein | AFS45420.1 |
| 35 | A964_0641 | LambdaSa04, minor structural protein | AFS45421.1 |
| 36 | A964_0642 | LambdaSa04, holin | AFS45422.1 |
| 37 | A964_0643 | N-acetylmuramoyl-L-alanine amidase, family 4 | AFS45423.1 |
| 38 | A964_0644 | LambdaSa04, site-specific recombinase resolvase | AFS45424.1 |
| 39 | A964_0645 | LambdaSa04, site-specific recombinase resolvase | AFS45425.1 |
| 40 | A964_0834 | *metK*, S-adenosylmethionine synthetase | AFS45606.1 |
| 41 | A964_0931 | Cdd, cytidine deaminase | AFS45701.1 |
| 42 | A964_0932 | *bmpA*, ABC superfamily ATP binding cassette transporter, binding protein | AFS45702.1 |
| 43 | A964_0937 | Ldh, L-lactate dehydrogenase | AFS45707.1 |
| 44 | A964_1009 | *ftsK*, FtsK/SpoIIIE family protein | AFS45777.1 |
| 45 | A964_1018 | *carB*, carbamoyl phosphate synthase large subunit | AFS45786.1 |
| 46 | A964_1019 | *carA*, carbamoyl phosphate synthase small subunit | AFS45787.1 |
| 47 | A964_1020 | *pyrB*, aspartate carbamoyltransferase | AFS45788.1 |
| 48 | A964_1021 | *pyrC*, dihydroorotase | AFS45789.1 |
| 49 | A964_1022 | *pyrE*, orotate phosphoribosyltransferase | AFS45790.1 |
| 50 | A964_1023 | *pyrF*, orotidine 5'-phosphate decarboxylase | AFS45791.1 |
| 51 | A964_1054 | NADPH-dependent FMN reductase domain-containing protein | AFS45822.1 |
| 52 | A964_1117 | *uraA*, uracil permease | AFS45882.1 |
| 53 | A964_1237 | Idi, isopentenyl pyrophosphate isomerase | AFS46002.1 |
| 54 | A964_1264 | surface antigen-like protein | AFS46029.1 |
| 55 | A964_1355 | *malE*, maltose/maltodextrin ABC transporter maltose/maltodextrin-binding protein | AFS46120.1 |
| 56 | A964_1393 | *smpB*, SsrA-binding protein | AFS46158.1 |
| 57 | A964_1429 | *bltL*, major facilitator transporter | AFS46194.1 |
| 58 | A964_1430 | M20/M25/M40 family peptidase | AFS46195.1 |
| 59 | A964_1554 | *dhaK*, dihydroxyacetone kinase subunit DhaK | AFS46315.1 |
| 60 | A964_1555 | *dhaL*, dihydroxyacetone kinase family protein | AFS46316.1 |
| 61 | A964_1556 | PTS system mannnose-specific transporter subunit IIA | AFS46317.1 |
| 62 | A964_1557 | *glpF*, glycerol uptake facilitator protein | AFS46318.1 |
| 63 | A964_1719 | PRK05261, putative phosphoketolase | AFS46478.1 |
| 64 | A964_1720 | *ulaG*, L-ascorbate 6-phosphate lactonase | AFS46479.1 |
| 65 | A964_1730 | *araD*, L-ribulose-5-phosphate 4-epimerase | AFS46489.1 |
| 66 | A964_1732 | *sgbH*, 3-keto-L-gulonate-6-phosphate decarboxylase | AFS46491.1 |
| 67 | A964_1733 | *sgaA*, PTS system transporter subunit IIA | AFS46492.1 |
| 68 | A964_1735 | *sgaT*, PTS system ascorbate-specific transporter subunit IIC | AFS46494.1 |
| 69 | A964_1778 | *sfcA*, malate dehydrogenase | AFS46535.1 |
| 70 | A964_1779 | citrate, cation symporter (CCS) family protein | AFS46536.1 |
| 71 | A964_1824 | crr, PTS system glucose-specific transporter subunit IIABC | AFS46579.1 |
| 72 | A964_1906 | major facilitator family protein | AFS46657.1 |
| 73 | A964_1909 | cell-wall anchored surface adhesin | AFS46660.1 |
| 74 | A964_1917 | *udp*, uridine phosphorylase | AFS46668.1 |
| 75 | A964_1954 | *rpmF*, 50S ribosomal protein L32 | AFS46705.1 |
| 76 | A964_1973 | *argI*, ornithine carbamoyltransferase | AFS46724.1 |
| 77 | A964_2010 | *arcA*, arginine deiminase | AFS46761.1 |
