# Supplementary material for: CRISPR-dependent endogenous gene regulation is required for virulence in piscine Streptococcus agalactiae
Source: Emerg Microbes Infect. 2021 Nov 12;10(1):2113–24. doi: 10.1080/22221751.2021.2002127 (PMC8592606; doi:10.1080/22221751.2021.2002127)
Supplement: Table_S3.docx [file TEMI_A_2002127_SM5757.docx]

Table S3 Calculation of LD_50_ for zebrafish

| Dose of challenge CFU/(0.02mL) | Number of death/total | | |
| --- | --- | --- | --- |
|  | WT | ΔCRISPR | CΔCRISPR |
| 1×10^6^ | 11/11 | 8/11 | 11/11 |
| 1×10^5^ | 11/11 | 7/11 | 11/11 |
| 1×10^4^ | 9/11 | 5/11 | 8/11 |
| 1×10^3^ | 8/11 | 4/11 | 6/11 |
| 1×10^2^ | 5/11 | 2/11 | 4/11 |
| 1×10^1^ | 1/11 | 1/11 | 1/11 |
| LD_50_ value | 2.43×10^2^ | 1.72×10^4^ | 5.46×10^2^ |
