# Supplementary material for: CRISPR-dependent endogenous gene regulation is required for virulence in piscine Streptococcus agalactiae
Source: Emerg Microbes Infect. 2021 Nov 12;10(1):2113–24. doi: 10.1080/22221751.2021.2002127 (PMC8592606; doi:10.1080/22221751.2021.2002127)
Supplement: Table_S2.docx [file TEMI_A_2002127_SM5756.docx]

Table S2 Primers used in this study

| **Primer** | **Sequence (5′–3′)** | | **Function** |
| --- | --- | --- | --- |
| CRISPR-A | | AAAACGACGGCCAGTGAATTC GATGTCAACTCAGCACCTATTC | Construction of ΔcrRNA |
| CRISPR-B | | AAAAGAAGATCTCGTAGAAAATTTTCTTTTGA |  |
| CRISPR-C | | TTTCTACGAGATCTTCTTTTGACCTAACAAAAGGA |  |
| CRISPR-D | | CCGGGTACCGAGCTCGAATTCTCTCCCGAAATAACTCCAATG |  |
| CRISPR-F | | GGTTTCATCGCACGACTTAC | Verification of ΔcrRNA |
| CRISPR-R | | TGAGGGTAGTCCTTGAATGG |  |
| tracrRNA-A | | GTTTTTGAATTCTAAATATGCCCAGCTTTCCTT | Construction of ΔtracrRNA |
| tracrRNA-B | | TCCTAGTTAGTCACCCGGGTAATGTCATTGTTATAATTCCGACAT |  |
| tracrRNA-C | | ATTGTTTTAGTACCTGGAGGGAATACAAAATTATTATATGTTATTTTAGT |  |
| tracrRNA-D | | GTTTTTGTCGACTGCTCCCTCTCTTATCTTCC |  |
| tracrRNA-F | | GGACTGGATTAAAAATCACTGCC | Verification of ΔtracrRNA |
| tracrRNA-R | | TGCTCCCTCTCTTATCTTCC |  |
| *covR/S*-A | | AAAACGACGGCCAGTGAATTC GTTGTAAAGCGGTTGCCA | Construction of Δ*covR/S* |
| *covR/S*-B | | GTTGGTGTAG GAAAAATCCTTATGCTTAATG |  |
| *covR/S*-C | | AGGATTTTTC CTACACCAACCTTCTTATTCTT |  |
| *covR/S*-D | | CCGGGTACCGAGCTCGAATTC ACAGAAATCAAAAAATCACCAG |  |
| *covR/S*-F | | AAAAACCTGGTTGTACAGGATC | Verification of Δ*covR/S* |
| *covR/S*-R | | TATGACACGAGAAGAGTTGCTA |  |
| C*covR/S*-F | | GAGCTCGGTACCCGGGGATCCATTTTGGACAACGCATGT | Complementation of *covR/S* |
| C*covR/S*-R | | CAGGTCGACTCTAGAGGATCCATTGAAAAGTGACAGAGGACA |  |
| *sag0671*-A | | AAAACGACGGCCAGTGAATTCTATGATGGAAAAGTTGTTGTC | Construction of Δ*sag0671* |
| *sag0671*-B | | CTGAGTTTTC TTTACTTTTCCTAAATCCAAAT |  |
| *sag0671*-C | | GAAAAGTAAA GAAAACTCAGAGAAAGGCAAT |  |
| *sag0671*-D | | CCGGGTACCGAGCTCGAATTCTTTTCCTTTGAGATTCTTTTC |  |
| *sag0671*-F | | GCCTATGCGGTGCTTTAT | Verification of Δ*sag0671* |
| *sag0671*-R | | TTACTTTTGGAGCTACCTTCTT |  |
| C*sag0671*-F | | GAGCTCGGTACCCGGGGATCCCTCCTCAAAAAGAGCCTATC | Complementation of Δ*sag0671* |
| C*sag0671*-R | | CAGGTCGACTCTAGAGGATCCTTACTCGTATTTAGGCAACTG |  |
| *cylE* -F | | ATTTCCTATTCTCCTCCTGGC | Used for qRT-PCR |
| *cylE* -R | | CTCTTGATGCCATAATCCTTCTC |  |
| *covS*-F | | CGGACGAACAATACGCTTAGTAG | Used for qRT-PCR |
| *covS*-R | | CAGGACAGTTGCTTGGATACG |  |
| *covR*-F | | AATGCTGTGTCCAATCCTTCA | Used for qRT-PCR |
| *covR*-R | | GCTCGCTTCGTCTCGTTAG |  |
| *sag0671-*qF | | GTTGTTGCTTCATCAGCGTGA | Used for qRT-PCR |
| *sag0671-*qR | | CAGCCTATGCGGTGCTTTATT |  |
| 16S rRNA-F | | CGACGATACATAGCCGACCT | Used for qRT-PCR |
| 16S rRNA-R | | CCGTCACTTGGTAGATTTTCC |  |
| IL-6-F | | CCACTTCACAAGTCGGAGGCTTA | Used for qRT-PCR |
| IL-6-R | | GCAAGTGCATCATCGTTGTTCATAC |  |
| IL-1β-F | | TCCAGGATGAGGACATGAGCAC | Used for qRT-PCR |
| IL-1β-R | | GAACGTCACACACCAGCAGGTTA |  |
| TNF-α-F | | AAGCCTGTAGCCCACGTCGTA | Used for qRT-PCR |
| TNF-α-R | | GGCACCACTAGTTGGTTGTCTTTG |  |
| β-actin-F | | TGACAGGATGCAGAAGGAGA | Used for qRT-PCR |
| β-actin-R | | GCTGGAAGGTGGACAGTGAG |  |
