# Supplementary material for: CRISPR-dependent endogenous gene regulation is required for virulence in piscine Streptococcus agalactiae
Source: Emerg Microbes Infect. 2021 Nov 12;10(1):2113–24. doi: 10.1080/22221751.2021.2002127 (PMC8592606; doi:10.1080/22221751.2021.2002127)
Supplement: Table_S1.docx [file TEMI_A_2002127_SM5755.docx]

Table S1 Bacterial strains and plasmids

| Strains and plasmids | Description | Source or reference |
| --- | --- | --- |
| Strains |  |  |
| *S. agalactiae* |  |  |
| GD201008-001 | A virulent strain of *S. agalactiae* isolated from tilapia with meningoencephalitis in China | Collected in our laboratory |
| ΔCRISPR | CRISPR deletion mutant in GD201008-001 | This study |
| Δ*cas9* | *cas9* gene deletion mutant in GD201008-001 | Collected in our laboratory |
| CΔCRISPR | ΔCRISPR complemented with CRISPR that integrated to chromosome | This study |
| Δ*tracrRNA* | tracrRNA deletion mutant in GD201008-001 | This study |
| CΔ*tracrRNA* | ΔtracrRNA complemented with tracrRNA that integrated to chromosome | This study |
| Δ*covR*/*S* | *covR*/*S* genes deletion mutant in GD201008-001 | This study |
| ΔCRISPR-*covR/S* | CRISPR and *covR*/*S* genes double deletion mutant in GD201008-001 | This study |
| CΔ*covR/S* | Δ*covR/s* complemented with vector pSET2::*covR/S* | This study |
| CΔ*covR/S*-ΔCRISPR | ΔCRISPR*-covR/S* complemented with vector pSET2::*covR/S* | This study |
| Δ*sag0671* | *sag0671* deletion mutant in GD201008-001 | This study |
| ΔCRISPR-*sag0671* | CRISPR and *sag0671* gene double deleted mutant in GD201008-001 | This study |
| WT+p*sag0671* | *sag0671* overexpression strain, WT complemented with vector pSET2:: *sag0671* | This study |
| *E. coli* DH5α | Production of recombinant plasmids | Invitrogen |
| *E. coli* M5 | Evaluation of BBB opening | Collected in our laboratory |
| Plasmids |  |  |
| pSET4S | Thermo-sensitive suicide vector for gene replacement in *Streptococcus* replication of pG+ host3and pUC19, *lacZ’*, Spc^r^ |  |
| pSET4S:: CRISPR | Recombinant vector with pSET4S background, designed for knockout of CRISPR; | This study |
| pSET4S::CCRISPR | Recombinant vector with pSET4S background, designed for complementary of CRISPR | This study |
| pSET4S::tracrRNA | Recombinant vector with pSET4S background, designed for knockout of tracrRNA | This study |
| pSET4S::CtracrRNA | Recombinant vector with pSET4S background, designed for complementary of tracrRNA | This study |
| pSET4S:: *covR*/*S* | Recombinant vector with pSET4S background, designed for knockout of *covR*/*S* | This study |
| pSET2 | Shuttle vector for gene complementary in *Streptococcus*; Spc^r^ | This study |
| pSET2:: *covR/S* | Complementation vector with pSET2 background, containing promoter followed by full-length *covR/S* ORF | This study |
| pSET2:: *sag0671* | Complementation vector with pSET2 background, containing promoter followed by full-length *sag0671* ORF | This study |
